# Molecular Damping Effect of Trace Additives Enhances Zinc Anode Stability Under High Depth of Discharge

**DOI:** 10.1002/advs.202507071

**Published:** 2025-07-28

**Authors:** Yue Li, Hao Xu, Xiaodong Li, Xi Lin, Hongyang Zhao, Yajuan Zhang, Kwun Nam Hui, Jinliang Li, Likun Pan

**Affiliations:** ^1^ Shanghai Key Laboratory of Magnetic Resonance School of Physics and Electronic Science Institute of Magnetic Resonance and Molecular Imaging in Medicine East China Normal University Shanghai 200241 P. R. China; ^2^ Faculty of Chemistry and Food Chemistry and Center for Advancing Electronics Dresden Technische Universität Dresden 01062 Dresden Germany; ^3^ Shanghai Key Laboratory of Hydrogen Science and Center of Hydrogen Science Shanghai Jiao Tong University Shanghai 200240 P. R. China; ^4^ School of Mechanical Engineering Shanghai Jiao Tong University Shanghai 200240 P. R. China; ^5^ Joint Key Laboratory of the Ministry of Education Institute of Applied Physics and Materials Engineering University of Macau Avenida da Universidade Taipa Macau SAR 519000 P. R. China; ^6^ Siyuan Laboratory Guangdong Provincial Engineering Technology Research Center of Vacuum Coating Technologies and New Energy Materials Department of Physics, College of Physics & Optoelectronic Engineering Jinan University Guangzhou 510632 P. R. China

**Keywords:** aqueous zinc‐ion batteries, high depth of discharge, molecular damping effect, PSVE, trace additive

## Abstract

Resolving the severe issues such as electric field distortion, dendritic zinc growth, and uneven zinc deposition under high depths of discharge (DOD) has become a significant hurdle of the aqueous zinc‐ion batteries (ZIBs). To address these challenges, an interfacial regulation strategy is proposed based on the molecular damping effect, in which a trace amount of weakly adsorbing additive is employed to stabilize the Zn anode interface by mitigating energy shocks and ionic disturbances induced by electric field fluctuations. Trace perfluorinated PSVE (erfluoro‐3,6‐dioxa‐4‐methyloct‐7‐enesulphonyl fluoride) is introduced to the traditional ZnSO_4_ electrolyte to optimize Zn deposition behavior on the zinc anode. Thus, the Zn//Zn symmetric batteries exhibit a prolonged cycling lifespan of over 200 h, even when operated at a high DOD of 85.5%. Additionally, the NVO (Na_2_V_6_O_16_) cathodes coupled with Zn anodes and modified electrolyte present a more stable capacity retention, maintaining a capacity of 141.98 mAh g^−1^ after 1000 cycles. Similarly, the full batteries assembled with the same electrodes in a ZnSO_4_ electrolyte retain only 51.49 mAh g^−1^ capacity after the same conditions. This work highlights the potential of the molecular damping effect as a promising solution for improving high DOD performance in ZIBs.

## Graphical abstract

Inspired by the energy dissipation from damping effects, this work employs trace amounts of weakly adsorbing additives to form an adapted interface on the Zn anode. Unlike the water‐deficient interfaces formed by strongly adsorbing additives, this adapted weakly adsorbing interface enables the regulation of electric field disturbances at higher depths of discharge, thereby enhancing the performance of aqueous zinc‐ion batteries.

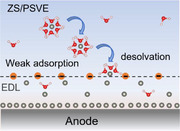

## 1 Introduction

Rechargeable aqueous zinc‐ion batteries (ZIBs) have garnered significant attention as a promising alternative to lithium‐ion batteries due to their inherent advantages, including high theoretical capacity and intrinsic safety.^[^ ^1^ ^]^ Moreover, ZIBs usually employ aqueous electrolytes, reducing the risk of toxic leaks and improving environmental sustainability.^[^ ^2^ ^]^ These features make ZIBs particularly attractive for large‐scale energy storage applications, including flexible energy storage and renewable energy integration. Despite their advantages, severe dendrite formation and electrode/electrolyte interface instability can lead to short circuits and significant degradation of the zinc anode interface.^[^ ^3^ ^]^ These issues present substantial challenges to the safety and lifespan of ZIBs. Additionally, side reactions such as hydrogen evolution can also lead to battery swelling and other detrimental effects.^[^ ^4^ ^]^ Especially due to the more intense electric field changes and the increased zinc ion concentration gradient, these side reactions become more pronounced under high depth of discharge conditions (DOD).^[^ ^5^ ^]^

When the Zn utilization of the anode is high (> 30%), severe Zn redistribution and interfacial heterogeneity exacerbate dendrite growth and side reactions, leading to electrode degradation.^[^ ^6^ ^]^ A large amount of metallic Zn is stripped, causing a rapid decline in Zn^2+^ concentration within the system under high DOD. This results in a sharp increase in the local electric field at the electrode surface, leading to a severe mismatch between electrochemical reaction kinetics and mass transport rates, which triggers a series of interface issues.^[^ ^7^ ^]^ Ultimately, these effects cause imbalanced platting/stripping processes, passivation of the active area, and irreversible capacity loss, significantly limiting the effective utilization and cycling life of the battery under high DOD.^[^ ^8^ ^]^ Therefore, maintaining electric field uniformity and structural stability at the Zn anode interface under such dramatic electric field variations remains a core scientific challenge for advancing ZIBs toward high‐energy density applications.^[^ ^9^ ^]^ To address these challenges, previous studies have attempted to stabilize the interface using water‐in‐salt systems,^[^ ^10^ ^]^ gel electrolytes,^[^ ^11^ ^]^ or strongly adsorbing additives^[^ ^12^ ^]^ that aim to construct dense interfacial layers to “shield” side reactions.^[^ ^13^ ^]^ While effective to some extent, such strategies often suffer from compromised ion transport, limited adaptability to different discharge conditions, and a lack of long‐term interfacial responsiveness.^[^ ^14^ ^]^ In particular, under high depths of discharge, where electric field fluctuations and mass transport demands are more intense, strongly adsorbed layers may hinder interfacial ion exchange and exacerbate polarization.

Inspired by the physical concept of damping, which describes the dissipation of excess mechanical energy through resistance or friction in dynamic systems,^[^ ^15^ ^]^ we propose a new interfacial regulation strategy that mimics this damping behavior at the electrochemical level. Instead of rigidly locking the interface through strong adsorpted additive, we introduce a trace amount of weakly adsorbing additive (PSVE) to establish a molecular damping effect on the surface of the Zn anode. Previous studies^[^ ^16^ ^]^ have shown that weakly adsorbing species can effectively modulate interfacial environments without forming dense, passivating layers, supporting the feasibility of this approach. This “damping layer” does not hinder interfacial reactions but rather buffers rapid fluctuations in the local electric field and ion flux, mitigating energy shocks and concentration disturbances. Analogous to how damping systems stabilize mechanical motion by smoothing oscillations, the molecular damping effect stabilizes the interfacial microenvironment, maintaining both structural integrity and ion transport continuity. In addition, the molecular damping effect offers three key advantages for high DOD conditions: 1) Electric field mitigation: by adjusting the local dielectric environment, the additives reduce electric field spikes and inhibit dendritic growth. 2) Ion flux balance: trace additives stabilize ion migration in regions with rapid concentration gradients, preventing local accumulation. 3) Reconstructing the interfacial layer: a more stable interfacial layer can be formed through the coordination interaction between additive functional groups and the zinc anode, thereby enhancing the reversibility of Zn^2+^ transport.

Building upon the above concept, this work employs trace amounts of PSVE as a model weakly adsorbing additive to regulate Zn^2+^ deposition behavior and investigates its effects under high DOD conditions. PSVE was selected due to its unique perfluorinated structure and sulfonic acid group, which confer strong ionic conductivity and the ability to form stable, weakly adsorptive layers on the Zn anode surface. Through a combination of COMSOL simulations and electrochemical testing, we demonstrate that trace PSVE significantly improves the uniformity of the electric field distribution on the zinc anode, suppresses tip effects, and promotes the formation of a Zn–F rich interface layer, enhancing overall interface stability. As a result, Zn//Zn symmetric batteries achieve stable cycling for more than 200 h at a high DOD of 85.5%. Furthermore, Cu//Zn half‐batteries maintain an ultrahigh coulombic efficiency (CE) of 99.9% over 350 cycles, indicating excellent reversibility of Zn plating/stripping. Most notably, the practical NVO//Zn full cell demonstrates a substantial improvement in long‐term performance, maintaining a capacity of 141.98 mAh g^−1^ after 1000 cycles with the ZS/PSVE electrolyte, which is significantly higher than the 51.49 mAh g^−1^ obtained with the ZS electrolyte under the same conditions.

## 2 Results and Discussion

**Figure 1:**
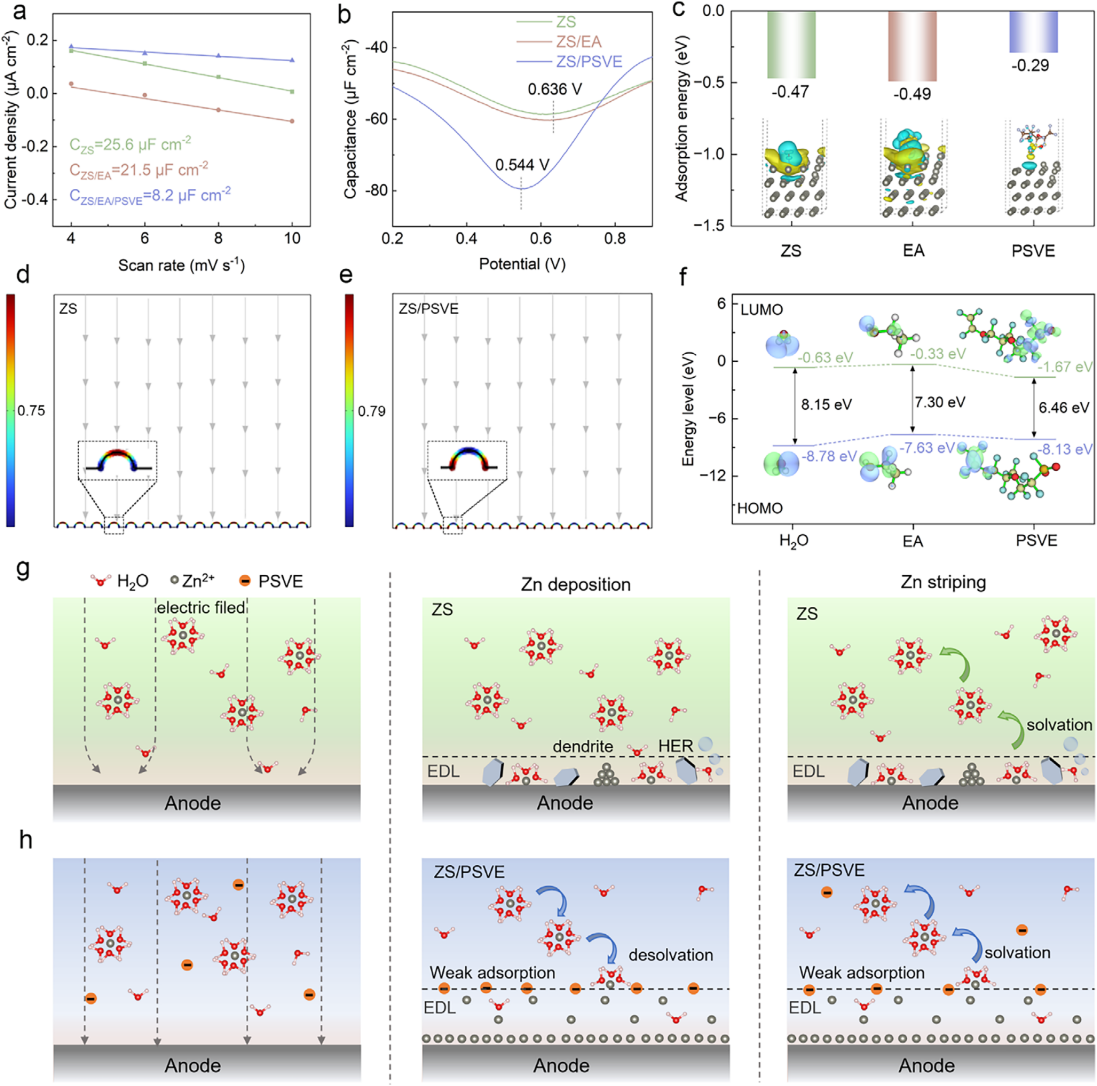
a) The electric double layer capacitance (EDLC) analyses of Zn anode in the ZS, ZS/EA, ZS/PSVE electrolytes. b) The alternating current voltammetries of the Zn anodes in the ZS, ZS/EA, ZS/PSVE electrolytes. c) The calculated adsorption energy of the H_2_O, EA, PSVE on the ZN (002) crystal plane. Simulated electric field distributions on the Zn in different electrolytes of d) the ZS electrolyte and e) the ZS/PSVE electrolyte. f) The HOMO‐LUMO energy level of H_2_O, EA, and PSVE additive. g) The Zn plating and striping diagram in the ZS electrolyte. h) The Zn plating and striping diagram in the ZS/PSVE electrolyte.a) The electric double layer capacitance (EDLC) analyses of Zn anode in the ZS, ZS/EA, ZS/PSVE electrolytes. b) The alternating current voltammetries of the Zn anodes in the ZS, ZS/EA, ZS/PSVE electrolytes. c) The calculated adsorption energy of the H_2_O, EA, PSVE on the ZN (002) crystal plane. Simulated electric field distributions on the Zn in different electrolytes of d) the ZS electrolyte and e) the ZS/PSVE electrolyte. f) The HOMO‐LUMO energy level of H_2_O, EA, and PSVE additive. g) The Zn plating and striping diagram in the ZS electrolyte. h) The Zn plating and striping diagram in the ZS/PSVE electrolyte.

The effectiveness of the molecular damping strategy and its influence on interfacial behavior were systematically demonstrated through experimental analysis, COMSOL simulation, and density functional theory (DFT) calculations, as shown in **Figure** **1**. The introduction of PSVE as an electrolyte additive leads to a significant reduction in the electrical double‐layer capacitance (EDLC), as evidenced by the decrease from 25.6 µF cm^−2^ in the ZS electrolyte to 8.2 µF cm^−2^ in the ZS/PSVE electrolyte, as shown in Figure 1a and Figure S1 (Supporting Information). This decrease suggests a fundamental rearrangement of the interfacial electric field, likely due to the interaction of PSVE and the zinc anode, thereby affecting the effective specific surface area of the anode.^[^ ^17^ ^]^ The experimental section shows the transformation equation between capacitance and current density. Moreover, Figure 1b displays that the potential of zero charge (PZC) shifts from 0.636 to 0.544 V with the addition of PSVE, indicating a modification interface of the anode, which aligns with the hypothesis of a more stable interface on the Zn anode.^[^ ^18^ ^]^ Notably, the interfacial electric field distribution on the Zn anode remains largely unaffected by ethanol (EA) as a co‐solvent, which is reflected in the minimal changes observed in the value of EDLC and ACV. From the DFT calculation results in Figure 1c, the adsorption energy of −0.29 eV indicates that PSVE will spontaneously adsorb onto the Zn (002) surface. However, due to its significant steric hindrance and weak polarization obtained from the electrostatic potential (ESP) mapping in Figure S2 (Supporting Information) PSVE shows a lower adsorption energy on the Zn (002) crystal plane compared to H_2_O (−0.47 eV) and the EA (−0.49 eV). This suggests that the observed electrochemical improvements are not due to simple, stronger, self‐adsorption‐driven interfacial stabilization of the additive on the zinc anode, but rather an indirect effect stemming from the weak adsorption of the additive, which modulates the electrostatic environment and charge transfer on the interface.

In contrast to the shielding effect induced by a strongly adsorbed and water‐depleted interface, the weakly adsorbed PSVE interface establishes a molecular damping mechanism that regulates interfacial electric field distribution and facilitates a more uniform electrochemical environment. COMSOL multiphysics simulations were carried out to explore how the molecular damping effect of PSVE influences the interfacial electric field and Zn^2+^ distribution during deposition. As shown in Figure 1d,e, the electric field tends to concentrate at surface protrusions on the anode, leading to uneven Zn^2+^ deposition in the ZS electrolyte.^[^ ^19^ ^]^ When trace amounts of PSVE are introduced into the electrolyte, the electric field becomes significantly more uniform across the Zn surface, effectively mitigating the tip effect. This modulation, attributed to the weakly adsorbed PSVE interface, also facilitates a higher local Zn^2+^ concentration on the surface of the zinc anode, enabling more uniform and rapid deposition (Figure S3, Supporting Information). In addition, the energy gaps between the highest occupied molecular orbital (HOMO) and the lowest unoccupied molecular orbital (LUMO) were calculated to be 6.46 eV for PSVE, 8.15 eV for H_2_O, and 7.30 eV for EA in Figure 1f. The relatively narrow energy gap of PSVE, along with simulation results, suggests that this weakly adsorbing molecule not only helps regulate electric field uniformity but also facilitates electron transport.^[^ ^20^ ^]^ These theoretical insights are further illustrated in a schematic diagram, which contrasts the interfacial electric field distribution and dendrite growth in the ZS electrolyte with and without PSVE. In the conventional ZS electrolyte (Figure 1g), Zn^2+^ tends to accumulate at sites with higher curvature, exacerbating dendrite growth.^[^ ^21^ ^]^ During Zn stripping, the desolvation process becomes highly heterogeneous, as Zn^2+^ solvation shells reform unevenly, further destabilizing the interface.^[^ ^22^ ^]^ In contrast, the weakly adsorbed PSVE molecular damping layer (Figure 1h), does not block ion transport but instead buffers electric field fluctuations and promotes a more uniform Zn^2+^ concentration distribution. As a result, dendrite formation is effectively inhibited, enabling smooth and uniform Zn deposition, thereby improving cycling stability under deep–discharge conditions.

**Figure 2:**
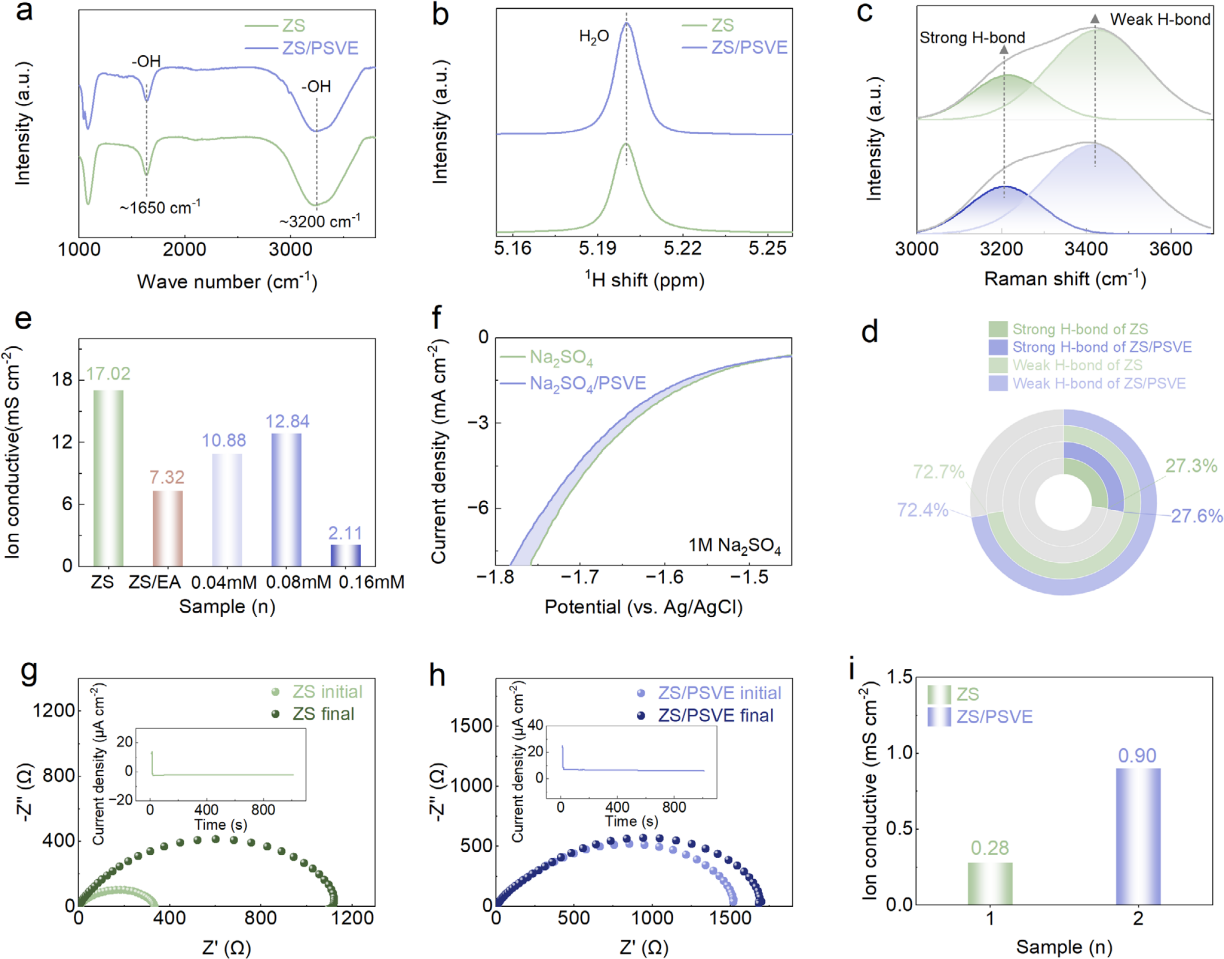
a) FTIR spectra, b) ^1^H NMR spectra of ZS and ZS/PSVE electrolytes. c) Raman spectra and d) the corresponding percentages of the strong H‐bond and weak H‐bond of ZS and ZS/PSVE electrolytes. e) The ionic conductivities of the ZS, ZS/EA, and ZS/PSVE electrolytes with different PSVE concentrations of 0.04, 0.08, and 0.16 mm. f) The LSV curves at 1 mV s^−1^ in the NaSO_4_ electrolytes with/without the PSVE additive. EIS of Zn//Zn symmetric batteries tested before and after CA testing in g) the ZS electrolyte and h) the ZS/PSVE electrolyte. i) The calculated Zn^2+^ transfer number.a) FTIR spectra, b) ^1^H NMR spectra of ZS and ZS/PSVE electrolytes. c) Raman spectra and d) the corresponding percentages of the strong H‐bond and weak H‐bond of ZS and ZS/PSVE electrolytes. e) The ionic conductivities of the ZS, ZS/EA, and ZS/PSVE electrolytes with different PSVE concentrations of 0.04, 0.08, and 0.16 mm. f) The LSV curves at 1 mV s^−1^ in the NaSO_4_ electrolytes with/without the PSVE additive. EIS of Zn//Zn symmetric batteries tested before and after CA testing in g) the ZS electrolyte and h) the ZS/PSVE electrolyte. i) The calculated Zn^2+^ transfer number.

Given that trace amounts of PSVE regulate the interfacial environment via weak adsorption and electric field damping, its effect on Zn^2+^ solvation shell is expected to be negligible. This assumption will be further validated through in‐depth spectroscopic characterizations. Fourier‐transform infrared (FTIR) spectra in **Figure** **2**a show minimal changes in the O–H stretching (≈3200 cm^−1^) and bending (≈1650 cm^−1^) vibrations between ZS and ZS/PSVE electrolytes, suggesting that trace PSVE does not significantly alter the bulk hydrogen‐bonding network of water. Similarly, Figure 2b presents the ^1^H nuclear magnetic resonance (NMR) spectra, where the chemical shift of water protons remains nearly unchanged, indicating that trace PSVE does not directly interact with solvent molecules or Zn^2+^. The nearly unaltered ─OH vibration peak in the Raman spectra (Figure 2c) provides additional evidence for this conclusion. Subsequent analysis of Raman spectra, deconvoluted into strong and weak H‐bond interactions, reveals that the relative proportions of H‐bonds remain essentially unchanged after PSVE modification.^[^ ^23^ ^]^ In the pristine ZS electrolyte, the percentages of strong and weak H‐bonds are 27.3% and 72.7%, respectively. After introducing trace PSVE, these percentages remain nearly unchanged, shifting to 27.6% for strong H‐bonds and 72.4% for weak H‐bonds (Figure 2d). According to the results, the structure of the Zn^2+^ solvation remains unchanged upon the addition of trace PSVE. Additionally, the minimal changes of the FTIR spectra (Figure S4, Supporting Information) and the Raman spectrum (Figure S5, Supporting Information) between the ZS and ZS/EA electrolytes suggest that the primary role of EA in the system is to act as a co‐solvent to enhance the solubility of hydrophobic additives, without interfering with the solvation structure. These results collectively show that trace PSVE does not significantly alter the overall chemical environment or molecular structure of the electrolyte. However, it influences the interface of the anode through an alternative interfacial mechanism through weak adsorption.

Despite the minimal effect on bulk solvation properties, PSVE effectively regulates the ionic conductivity of the electrolytes. As shown in Figure S6 (Supporting Information) and Figure 2e, adding EA to the ZS electrolyte reduces the conductivity to 7.32 mS cm^−2^ less than that of the ZS electrolyte of 17.02 mS cm^−2^ due to the increased viscosity, which limits ion mobility. Interestingly, the introduction of trace amounts of PSVE leads to a non‐monotonic trend: the conductivity increases with 0.04 and 0.08 mm PSVE to 10.88 and 12.84 mS cm^−2^, and then decreases again at 0.16 mm to 2.11 mS cm^−2^. This suggests that low concentrations of PSVE may facilitate ion transport. However, excess PSVE may introduce structural heterogeneity or increase viscosity, thereby offsetting these benefits. This enhancement is primarily attributed to interfacial regulation: the weakly adsorbing PSVE molecules form a dynamic, non‐blocking layer on the electrode surface, which modulates the local electric field, smooths ion migration pathways, and reduces charge transfer resistance.^[^ ^24^ ^]^ As a result, ion mobility near the interface is improved, leading to higher conductivity without disrupting the electrolyte structure.^[^ ^25^ ^]^ Notably, Figure 2f presents linear sweep voltammetry (LSV) curves of electrolytes with/without the PSVE additive. The result shows a slight improvement in HER onset potential in the PSVE‐modified electrolyte because hydrogen bonding remains rarely affected. Rather, this improvement is likely due to a more homogeneous interfacial distribution of Zn^2+^ ions, which effectively suppresses localized overpotential fluctuations that could otherwise promote the hydrogen evolution reaction (HER).^[^ ^26^ ^]^ Figure 2g–i provides additional proof of the interfacial optimization for the PSVE. A dramatic increase of the Zn^2+^ transfer number from 0.28 of the ZS electrolyte to 0.90 of the ZS/PSVE electrolyte indicates that PSVE strongly favors Zn^2+^‐dominant charge transport, effectively reducing polarization effects.^[^ ^27^ ^]^ The consistency among these findings supports the notion that PSVE does not function through traditional, stronger adsorption‐driven mechanisms but instead induces a regulation of the interface restructuring process by weak adsorption, enhancing Zn^2+^ conduction while simultaneously mitigating parasitic side reactions such as HER.

**Figure 3:**
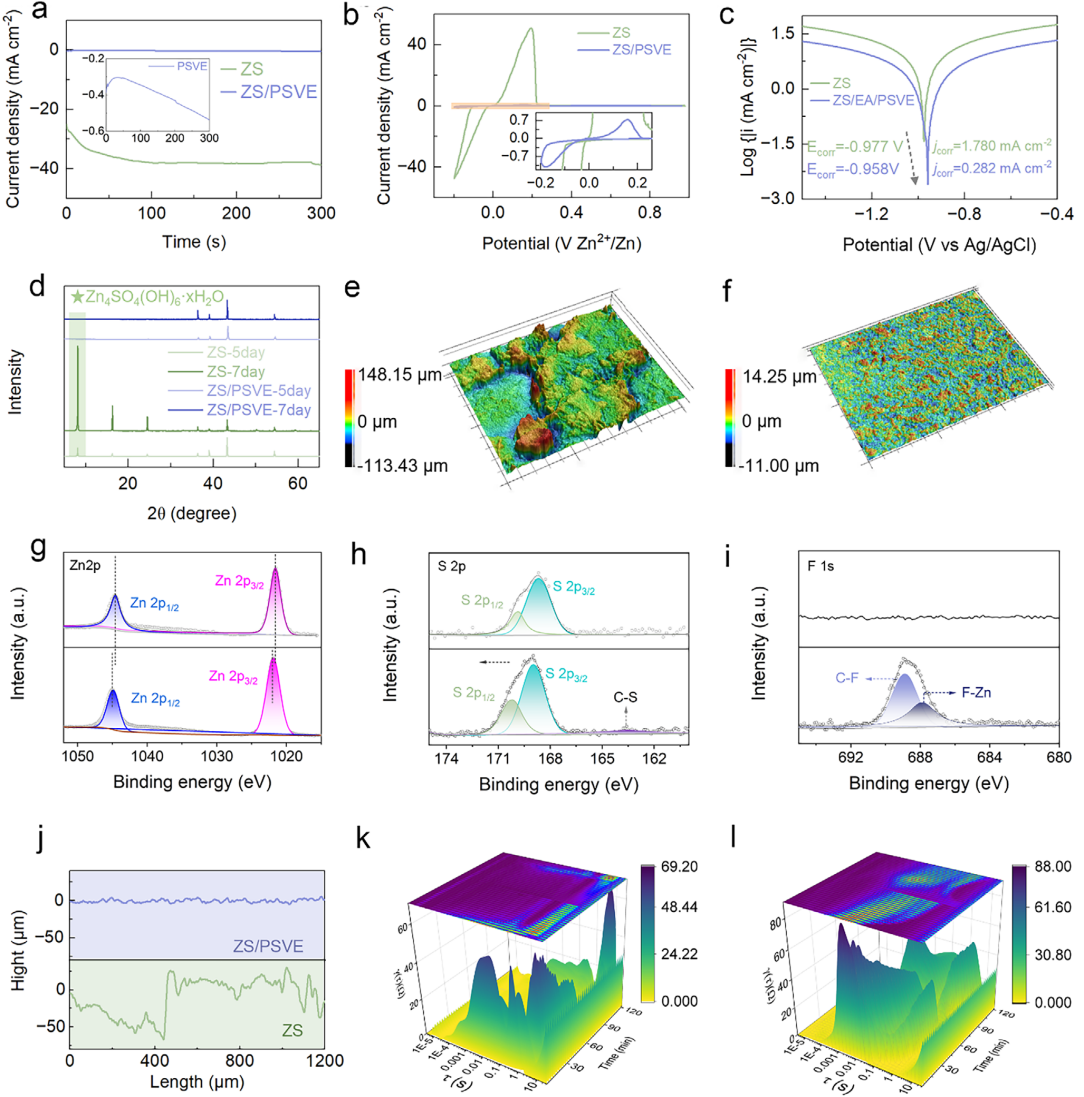
a) CA curves and b) CV curves of Cu//Zn batteries. c) the Tafel profiles of the three‐electrode system tested in the ZS and ZS/PSVE electrolytes. d) The corresponding XRD patterns of Zn foils after soaking. The laser microscopy images of Zn anodes after plating/stripping for 20 cycles in e) the ZS electrolyte and f) the ZS/PSVE electrolytes at the 5 mA cm^−2^@5 mAh cm^−2^. The X‐ray photoelectron spectroscopy XPS spectra of g) Zn 2p, h) S 2p, i) F 1s, of the Zn anodes in the ZS (up) and ZS/PSVE (down) electrolytes after cycling for 20 cycles at the 5 mA cm^−2^@5 mAh cm^−2^. j) The surface height profiles of Zn anodes corresponding to laser microscopy results. The DRT plots of Zn//Zn symmetric cells during the first plating and stripping using k) the ZS and l) the ZS/PSVE electrolyte.a) CA curves and b) CV curves of Cu//Zn batteries. c) the Tafel profiles of the three‐electrode system tested in the ZS and ZS/PSVE electrolytes. d) The corresponding XRD patterns of Zn foils after soaking. The laser microscopy images of Zn anodes after plating/stripping for 20 cycles in e) the ZS electrolyte and f) the ZS/PSVE electrolytes at the 5 mA cm^−2^@5 mAh cm^−2^. The X‐ray photoelectron spectroscopy XPS spectra of g) Zn 2p, h) S 2p, i) F 1s, of the Zn anodes in the ZS (up) and ZS/PSVE (down) electrolytes after cycling for 20 cycles at the 5 mA cm^−2^@5 mAh cm^−2^. j) The surface height profiles of Zn anodes corresponding to laser microscopy results. The DRT plots of Zn//Zn symmetric cells during the first plating and stripping using k) the ZS and l) the ZS/PSVE electrolyte.

A weakly adsorptive interface modulates Zn^2+^ deposition behavior, which is crucial for Zn anode stability. Chronoamperometry (CA) testing was performed to differentiate these deposition behaviors by analyzing the temporal evolution of deposition current at a constant overpotential of −150 mV. As shown in **Figure** **3**a, the current density steadily increases within 100 s in the ZS electrolyte, indicating that Zn^2+^ ions initially undergo 2D surface diffusion‐controlled deposition and migrate laterally to energetically favorable sites.^[^ ^28^ ^]^ This lateral movement promotes Zn^2+^ aggregation at high‐curvature regions, where local electric field enhancement accelerates Zn nucleation, eventually leading to uneven growth and dendrite formation (see Figure S7a, Supporting Information).^[^ ^29^ ^]^ In contrast, with the introduction of trace PSVE to the electrolyte, the current density remains nearly stable over time, suggesting a sustained 3D diffusion‐controlled process (see Figure S7b, Supporting Information). The different Zn deposition behavior could also be confirmed by the SEM images in Figure S8 (Supporting Information). This indicates that Zn^2+^ ions experience enhanced bulk ion transport and uniform nucleation, facilitating bottom‐up deposition on the Zn anode. The fast Zn^2+^ transfer and stable interfacial electric field contribute to a more compact and homogeneous Zn deposition layer, effectively mitigating dendritic growth and improving electrode stability.^[^ ^30^ ^]^ Subsequently, cyclic voltammetry (CV) testing evaluates the Zn^2+^/Zn redox process. Figure 3b shows that Cu//Zn batteries using the ZS/PSVE electrolyte display a lower nucleation overpotential of 76 mV than those using the ZS electrolyte, thus favoring more uniform and controllable Zn plating in the ZS/PSVE electrolyte.^[^ ^31^ ^]^ The PSVE is proposed to form an interfacial layer on the zinc surface through weak adsorption, which inhibits direct electron and ion transfer at the interface, resulting in a reduced current response. While uniform Zn deposition is crucial for stable cycling, the long‐term cycling performance of Zn anodes also depends on the resistance to corrosion. As shown in Figure 3c, Tafel polarization testing results reveal that the corrosion current density of Zn foils in the ZS/PSVE electrolyte is 0.282 mA cm^−2^, lower than that in the ZS electrolyte (1.780 mA cm^−2^). Moreover, Zn foils in the ZS/PSVE electrolyte show a higher corrosion potential (vs Ag/AgCl) of −0.958 V than that in the ZS electrolyte (−0.977 V). These results indicate that Zn anodes have stronger corrosion resistance in the ZS/PSVE electrolyte compared with the ZS electrolyte.^[^ ^32^ ^]^

These corrosion characteristics were further examined by immersing Zn foils in different electrolytes for 7 days to analyze byproduct formation and surface degradation. SEM images (Figure S9, Supporting Information) clearly illustrate the progressive formation and growth of byproducts on Zn foils immersed in the ZS electrolyte over time. After 7 days of immersion, these byproducts become more pronounced, with their characteristic peaks of Zn_4_SO_4_(OH)_6_·*x*H_2_O (ZHS) appearing distinctly in the XRD patterns (Figure 3d). In contrast, the ZHS by‐product is largely suppressed in the ZS/PSVE electrolyte, indicating that the PSVE plays a crucial role in maintaining the long‐term cycling stability of Zn anodes. After 20 cycles of plating/stripping, the Zn anode coupled with the ZS electrolyte exhibits significant roughness on the surface, with a height variation of ≈260 µm (Figure 3e). In contrast, the Zn anode in the ZS/PSVE electrolyte maintains a uniform and smooth surface morphology (Figure 3f). Surface topography mapping further confirms severe corrosion, dendritic growth, and byproduct formation in the ZS electrolyte, which compromises the structural integrity of the Zn anode and leads to inferior cycling stability (see the surface profiles in Figure 3j). X‐ray photoelectron spectroscopy (XPS) studies were used to further examine the surface composition of the anode (Figure S10, Supporting Information). A new peak at 688.78 eV, attributed to F 1s, emerged on the surface of the Zn foil cycled in ZS/PSVE electrolyte in comparison to the Zn foil cycled in the ZS electrolyte. The shifted Zn 2 p_3/2_ and Zn 2p_1/2_ peaks are located at 1021.88 and 1044.98 eV in Figure 3g, indicating the presence of Zn^2+^ valence states on the surface of the Zn anode, which can be related to the Zn─F bond.^[^ ^33^ ^]^ This is consistent with the new C─–S peak in the S 2p spectrum, which originates from the C─S bond in the PSVE, indicating that PSVE is bonded to the Zn anode (Figure 3h).^[^ ^34^ ^]^ The presence of the F ions in distinct chemical environments is indicated by the deconvolution of the F 1s peak in Figure 3i into two component peaks.^[^ ^35^ ^]^ The binding energy at 687.98 eV represents the Zn─F bond. The deconvoluted F 1s signal at 688.88 eV can be attributed to the C─F bond in the PSVE.^[^ ^36^ ^]^ These results clearly show that steady Zn^2+^ deposition is encouraged by the consistent weak adsorption layer of the PSVE on the interface of the Zn anode during charging and discharging.

Then, in situ electrochemical impedance spectroscopy (EIS) analyses were employed to monitor the impedance evolution of the Zn anode during the first plating and stripping (Figure S11, Supporting Information). In the ZS electrolyte, the initial Zn^2+^ nucleation during plating exhibits a relatively high interfacial resistance. As plating undergoes, Zn^2+^ preferentially deposits in regions with locally intensified electric fields, resulting in a rapid decrease in interfacial resistance, as reflected by a sudden drop in impedance in the Nyquist plot. This process promotes highly anisotropic dendrite growth and uneven deposition. During Zn stripping, the localized depletion of Zn^2+^ slows lower ion diffusion, further exacerbating interfacial instability. Meanwhile, by‐products such as ZHS may gradually accumulate on the surface of the anode, forming an interfacial contamination layer that impedes Zn^2+^ transport, thereby increasing interfacial resistance (Figure S11a, Supporting Information). In contrast, in the PSVE‐containing electrolyte, the Zn^2+^ deposition process is modulated by the interfacial electric field, leading to more uniform Zn plating. The absence of excessive local electric field intensification prevents abrupt changes in deposition behavior. As a result, the impedance curve remains relatively stable throughout the deposition process, indicating a well‐regulated and consistent Zn plating/stripping interface (Figure S11b, Supporting Information). To resolve individual relaxation processes from the EIS data, the distribution of relaxation times (DRT) method was applied to decompose the impedance spectra and elucidate the underlying kinetic contributions. The DRT analysis reveals a fundamental difference in Zn deposition mechanisms between these two electrolytes. In the ZS electrolyte (Figure 3k), the relaxation behavior is dominated by a higher time constant (10^−1^–10^0^ s), indicating that Zn^2+^ deposition is primarily governed by a diffusion‐controlled process,^[^ ^37^ ^]^ leading to concentration polarization and uncontrolled dendritic growth. In contrast, the Zn anode in the ZS/PSVE electrolyte exhibits a dominant relaxation feature within 10^−2^–10^−1^ s, corresponding to a charge transfer‐controlled process (Figure 3l).^[^ ^38^ ^]^ This provides direct evidence that the presence of the PSVE effectively shifts the deposition behavior from a diffusion‐controlled to a charge transfer‐controlled process. Such regulation suppresses dendrite formation by promoting more uniform Zn plating and stripping while also highlighting the role of the weakly adsorptive PSVE damping layer in enhancing charge transfer kinetics. The in situ optical microscopy images to directly visualize the Zn deposition behavior in the Figure S12 (Supporting Information). In the ZS electrolyte, pronounced dendritic growth was observed over time, accompanied by increased surface roughness. By contrast, the PSVE‐containing electrolyte effectively suppressed dendrite formation on the zinc surface, resulting in a more uniform and stable deposition morphology. Therefore, the trace PSVE plays a crucial role in stabilizing the interface between the anode and electrolyte, reducing interfacial resistance fluctuations, and improving the long‐term cycling stability of the Zn anode.

**Figure 4:**
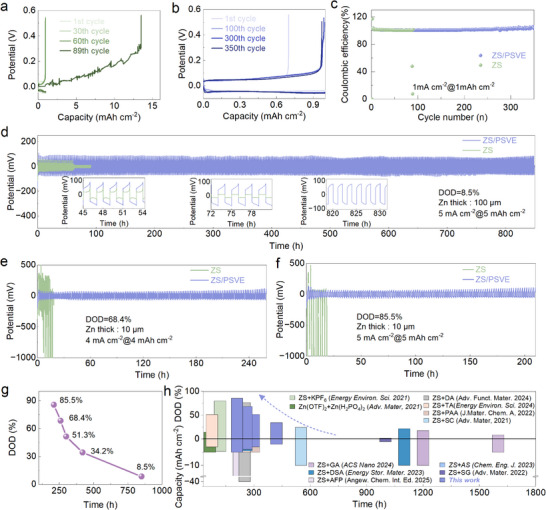
a) The voltage profiles of the Cu//Zn battery at different cycles in the ZS electrolyte. b) The voltage profiles of the Cu//Zn battery at different cycles in the ZS/PSVE electrolyte. c) The CE of Cu//Zn batteries in the different electrolytes. Long‐term galvanostatic cycling of symmetrical Zn//Zn batteries at high DOD: d) 8.5%, e) 68.4%, and f) 85.5%. g) DOD summary of Zn//Zn batteries fabricated by the ZS/PSVE electrolyte. h) Comparison of cyclic reversibility in the recent reports, the cited references have been listed in the Supporting Information.a) The voltage profiles of the Cu//Zn battery at different cycles in the ZS electrolyte. b) The voltage profiles of the Cu//Zn battery at different cycles in the ZS/PSVE electrolyte. c) The CE of Cu//Zn batteries in the different electrolytes. Long‐term galvanostatic cycling of symmetrical Zn//Zn batteries at high DOD: d) 8.5%, e) 68.4%, and f) 85.5%. g) DOD summary of Zn//Zn batteries fabricated by the ZS/PSVE electrolyte. h) Comparison of cyclic reversibility in the recent reports, the cited references have been listed in the Supporting Information.

To further evaluate the impact of the electrolyte additive on electrochemical performance, various battery configurations, including Cu//Zn half‐batteries and Zn//Zn symmetric batteries, were assembled. The electrochemical performance of Cu//Zn batteries were compared using a conventional ZS, ZS/EA, ZS/PSVE containing 0.04, 0.08, and 0.16 m PSVE electrolytes under a current density of 1 mA cm^−2^ for an areal capacity of 1 mAh cm^−2^. As shown in **Figure** **4**a, the Cu//Zn battery using the conventional ZS electrolyte experiences a short circuit after 89 cycles, indicating severe Zn dendrite growth and electrode degradation. In contrast, Figure 4b demonstrates that the Cu//Zn batteries using the ZS/PSVE electrolyte remain stable during cycling, highlighting their enhanced durability. Furthermore, Figure 4c and Figure S13 (Supporting Information) show that the additive‐containing electrolyte could prolong the cycle time of Zn//Cu battery and the 0.08 mm PSVE addition represents the optimal concentration under the tested conditions, maintains a consistently higher C of 99.9% after 350 cycles. Symmetric Zn//Zn batteries were assembled and tested to provide a more direct assessment of dendrite suppression, interfacial stability, and cycling durability, especially under high DOD conditions. As shown in Figure 4d–f and Figures S14 and S15 (Supporting Information), the ZS/PSVE electrolyte significantly enhances the cycling stability of symmetric Zn//Zn cells compared to that with the ZS electrolyte under gradient current densities. Figure 4g further confirms the robustness of the ZS/PSVE system under varying depths of discharge (DOD), ranging from 8.5% to 85.5%. Notably, the ZS/PSVE‐based Zn//Zn cells exhibit outstanding cycling stability across all tested DOD levels. Even at an ultrahigh DOD of 85.5%, which typically imposes severe polarization on the Zn anode, the battery maintains stable cycling for over 200 h with minimal voltage hysteresis. In Figure 4h and Table S1 (Supporting Information) a comprehensive comparison with previously reported electrolytes highlights the superior performance of the ZS/PSVE system in terms of stable cycling duration at high DOD. Collectively, these results demonstrate the effectiveness of the PSVE additive in regulating interfacial behavior and promoting dendrite‐free Zn plating/stripping under harsh electrochemical conditions.

In addition, the influence of trace PSVE concentration (0.04, 0.08, and 0.16 mm) in the electrolyte on the performance of Zn//Zn symmetric batteries was investigated. As shown in Figure S16 (Supporting Information), the addition of a trace amount of PSVE significantly enhances the stability of Zn//Zn symmetric batteries. Specifically, under a current density of 5 mA cm^−2^@5 mAh cm^−2^, the 0.08 mm ZS/PSVE‐based batteries achieve markedly prolonged cycling times, maintaining stable voltage profiles over 850 h without short‐circuiting or severe polarization, whereas batteries using the ZS electrolyte fail much earlier due to dendritic growth and interfacial instability even for 60 h. Remarkably, adding just 0.04 mm PSVE to the ZS electrolyte significantly extends the lifespan of the batteries to over 600 h, highlighting the effectiveness of trace PSVE in regulating the interface between the electrolyte and the anode, likely by forming a weakly adsorptive damping layer that promotes uniform Zn^2+^ deposition and suppresses dendrite growth. However, increasing the concentration to 0.16 mm leads to a reduced lifespan of ≈300 h, possibly due to excessive interfacial adsorption that hinders ion transport or disrupts interfacial dynamics. Besides, the effect of the co‐solvent of EA on the electrochemical performance of electrolytes was studied. Figure S16 (Supporting Information) also shows that the lifespan of Zn//Zn symmetric battery will extend to 400 h with the ZS/EA electrolyte. However, compared to the Zn//Zn symmetric battery assembled with the ZS electrolyte, those with the ZS/EA electrolyte exhibit a nearly threefold increase in the overpotential, reaching ≈200 mV. Notably, with the introduction of a trace amount (0.08 mm) of PSVE, the overpotential of Zn//Zn symmetric battery is significantly reduced to 90 mV, while the cycling stability is doubled compared to that using the ZS/EA electrolyte. Such significant performance improvement comes from the higher Zn^2+^ transfer number and ionic conductivity provided by the intrinsic properties of the PSVE (Figure S6, Supporting Information; Figure 2e). To further validate the concentration‐dependent effect of PSVE, we conducted the XRD analysis on the Zn anode retrieved from Zn//Zn symmetric batteries operated in five different electrolytes, respectively. As shown in Figure S17 (Supporting Information), the intensity ratio of the (002) to (101) plane (I_002_/I_101_) serves as a quantitative indicator of the preferred Zn crystallographic orientation. A notable evolution in this ratio is observed across the samples, with values of 0.41 (ZS), 0.69 (ZS/EA), 0.89 (0.04 mm PSVE), 5.24 (0.08 mm PSVE), and 1.16 (0.16 mm PSVE). The markedly enhanced value of I_002_/I_101_ in the 0.08 m PSVE sample suggests a strong promotion of Zn (002) texture, which is known to suppress dendritic growth and improve cycling reversibility, further reinforcing the mechanistic role of PSVE in modulating Zn deposition behavior. These results demonstrate that the addition of the trace PSVE could enhance the stability of the Zn anode, particularly under high DOD conditions.

**Figure 5:**
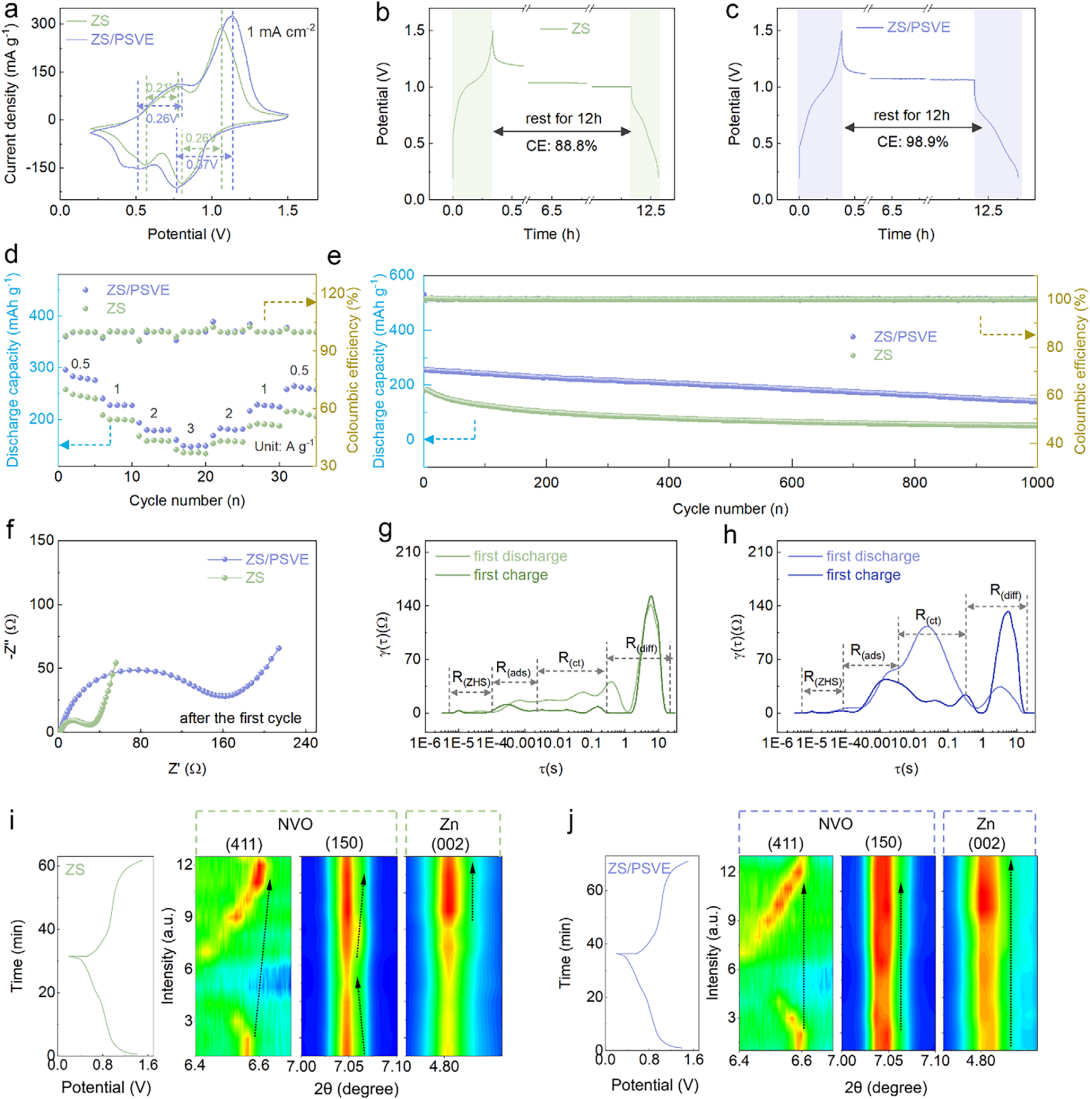
a) The CV curves of the (Na_2_V_6_O_16_) NVO//Zn batteries using the ZS and ZS/PSVE electrolytes. The self‐discharge curves of the NVO//Zn batteries resting for 12 h using b) the ZS electrolyte and c) the ZS/PSVE electrolyte. d) The rate performance of the NVO//Zn batteries using the ZS and ZS/PSVE electrolytes. e) The cycling performance NVO//Zn batteries using the ZS and ZS/PSVE electrolytes. f) EIS curves of NVO//Zn batteries using different electrolytes after the first cycle. The DRT curves of the NVO//Zn batteries using g) the ZS electrolyte and h) the ZS/PSVE electrolyte during the first discharge and charge process. In situ synchrotron XRD patterns of full NVO//Zn batteries during the first charge and discharge process in i) the ZS electrolyte and j) the ZS/PSVE electrolyte.a) The CV curves of the (Na_2_V_6_O_16_) NVO//Zn batteries using the ZS and ZS/PSVE electrolytes. The self‐discharge curves of the NVO//Zn batteries resting for 12 h using b) the ZS electrolyte and c) the ZS/PSVE electrolyte. d) The rate performance of the NVO//Zn batteries using the ZS and ZS/PSVE electrolytes. e) The cycling performance NVO//Zn batteries using the ZS and ZS/PSVE electrolytes. f) EIS curves of NVO//Zn batteries using different electrolytes after the first cycle. The DRT curves of the NVO//Zn batteries using g) the ZS electrolyte and h) the ZS/PSVE electrolyte during the first discharge and charge process. In situ synchrotron XRD patterns of full NVO//Zn batteries during the first charge and discharge process in i) the ZS electrolyte and j) the ZS/PSVE electrolyte.

To verify the practical application of the stabilized Zn anode, Na_2_V_6_O_16_//Zn full batteries were assembled using the ZS and ZS/PSVE electrolytes. As a normally used cathode material, the Na_2_V_6_O_16_ (NVO) was prepared by a hydrothermal method, which supports ion insertion and extraction.^[^ ^39^ ^]^ The XRD pattern (Figure S18, Supporting Information) and the SEM image (Figure S19, Supporting Information) can confirm the successful synthesis of NVO nanowires, showing distinct characteristic peaks of NVO. The CV curves in **Figure** **5**a demonstrate that the PSVE‐containing electrolyte enhances the redox potential gap (0.26 to 0.37 V, 0.21 to 0.26 V) for the NVO//Zn full batteries.The increased redox peak gap likely results from the formation of a protective interphase layer on the electrode surface, which introduces additional charge‐transfer resistance but contributes to enhanced interfacial stability and long‐term battery performance.^[^ ^40^ ^]^ Notably, the introduction of trace PSVE results in a substantial enhancement in self‐discharge resistance of the full batteries (Figure 5b,c). The NVO//Zn full battery utilizing the PSVE‐containing electrolyte retained 98.9% of its initial capacity after 12 h of rest, far exceeding the 88.8% retention observed in the battery using the ZS electrolyte. The excellent capacity retention of the NVO//Zn full battery indicates that the PSVE‐modulated interface on the Zn anode effectively mitigates side reactions and Zn^2+^ loss, particularly during long resting periods. The impact of PSVE on interfacial stability was well reflected in the rate performance of NVO//Zn full batteries (Figure 5d). The full battery with the ZS/PSVE electrolyte exhibits consistently higher specific capacities at all tested current densities from 0.5 to 3 A g^−1^. Moreover, when the current density returns to 0.5 A g^−1^, the battery recovers its original capacity, highlighting its reversibility and structural stability. Furthermore, the full battery assembled with the ZS/PSVE electrolyte exhibits an outstanding long‐term cycling stability, retaining a high reversible capacity of 141.98 mAh g^−1^ after 1000 cycles (Figure 5e). In contrast, the battery with the ZS electrolyte exhibits rapid capacity decay, retaining only 51.49 mAh g^−1^ under the same conditions.

In order to gain deeper insights into the origin of the enhanced full‐battery performance, a comprehensive mechanistic analysis was carried out. As shown in Figure 5f, the increased charge transfer resistance (R_ct_) after one cycle with the ZS/PSVE electrolyte implies the formation of a modified interface on the Zn anode, which may act as a protective barrier and contribute to improved long‐term cycling stability. Then, DRT analysis based on Nyquist plots during the first charge and discharge process was conducted in Figure 5g,h. Four separate relaxation times (τ_R_) are observed, each linked to intrinsic resistance (R_ZHS,_ 10^−^⁵–10^−^⁴ s), adsorption effects (R_ads_, 10^−3^–10^−2^ s), charge transfer dynamics (R_ct_, 10^−2^–10^−1^ s), and diffusion processes (R_diff_, 10^−1^–10^1^ s), respectively.^[^ ^41^ ^]^ The emergence of noticeable R_ads_ and R_ct_ after the first discharge process suggests that PSVE facilitates the formation of a compact interface between the electrolyte and electrode by the Zn‐F bond (related to the result in XPS, Figure 3i), which mitigates undesired reactions and slightly increases Zn^2+^ migration resistance.^[^ ^42^ ^]^ The batteries using the ZS/PSVE electrolyte exhibit not only a significantly shorter relaxation time for the diffusion process compared to that of the ZS electrolyte (3.16 s vs. 6.12 s of the R_diff_), but also a markedly smaller convolution peak area associated with migration impedance during the discharge process. This may reflect a more streamlined electrochemical pathway with reduced parasitic reactions, contributing to lower polarization and thus a lower observed redox potential.^[^ ^43^ ^]^ To evaluate the reversibility of the NVO cathode and the zinc anode, we performed in situ synchrotron XRD patterns during the first charge and discharge cycle. In the ZS electrolyte (Figure 5i), the (411) plane of the NVO cathodes shifts irreversibly to a higher degree after one cycle, indicating lattice contraction and structural irreversibility, thereby affecting the stability and reversibility of the NVO cathode. The peak of the (150) plane also shows significant variation in area during cycling, suggesting unstable structural behavior. These are the key factors causing battery capacity decay. In contrast, in the PSVE electrolyte (Figure 5j), the (411) plane could return to its original position after cycling, and the (150) plane remains stable, indicating improved structural reversibility and integrity. Moreover, the Zn (002) peak on the anode remains growing steadily in the ZS/PSVE electrolyte, suggesting more uniform and continuous Zn plating behavior. The growth of the (002) crystal plane could effectively inhibit the generation of dendrites and extend the battery lifespan. The ex situ SEM images of the NVO cathode after 1000 cycles in the PSVE electrolyte demonstrate a stable microstructure, with no apparent morphological degradation such as cracking or pulverization (Figure S20, Supporting Information). These observations suggest that the PSVE additive plays a protective role in preserving cathode architecture during extended cycling. Additionally, the stable performance of the full batteries at low N/P ratio demonstrates that the additive PSVE continues to enable stable cycling performance and high reversibility of the Zn anode even under these stringent conditions (Figure S21, Supporting Information).

## 3 Conclusion

In this study, we highlight a weakly adsorbed interfacial regulation strategy by introducing PSVE as a trace electrolyte additive to stabilize Zn anodes in aqueous ZIBs. Under high depth‐of‐discharge (DOD) conditions, PSVE effectively modulates the interfacial electric field and ion distribution through its weak adsorption and molecular damping effect, leading to uniform Zn deposition and enhanced reversibility of Zn plating/stripping. As a result, the PSVE‐containing electrolyte enables significant improvements in anode stability, evidenced by a reduced EDLC (8.2 µF cm^−2^), higher ionic conductivity (12.84 mS cm^−1^), and increased Zn^2+^ transference number (0.90), as well as the formation of a stable Zn–F interphase. The Zn//Zn symmetric cell demonstrates excellent cycling stability for over 200 h at a DOD of 85.5%, and the NVO//Zn full cell also shows markedly improved performance. This work not only advances the understanding of interfacial electrochemistry under deep–discharge regimes but also offers a promising route toward durable aqueous Zn–based batteries. This strategy may also be applicable to other metal systems, warranting further investigation into adapted interactions at the molecular scale.

## Conflict of Interest

The authors declare no conflict of interest.

## Supporting information



Supporting Information

## Data Availability

The data that support the findings of this study are available from the corresponding author upon reasonable request.
