# Supplementary material for: Molecular Damping Effect of Trace Additives Enhances Zinc Anode Stability Under High Depth of Discharge
Source: Adv Sci (Weinh). 2025 Jul 28;12(37):e07071. doi: 10.1002/advs.202507071 (PMC12499422; doi:10.1002/advs.202507071)
Supplement: Supplementary file 1 — Supporting Information [file ADVS-12-e07071-s001.docx]

**Supporting Information**

**Molecular Damping Effect of Trace Additives Enhances Zinc Anode Stability under High Depth of Discharge**

*Yue Li,^a^ Hao Xu,^b*^ Xiaodong Li,^b^ Xi Lin,^c^ Hongyang Zhao,^a^ Yajuan Zhang,^d^ Kwun Nam Hui,^e^ Jinliang Li,^f^* Likun Pan^a*^*

*^a^ Shanghai Key Laboratory of Magnetic Resonance, School of Physics and Electronic Science, Institute of Magnetic Resonance and Molecular Imaging in Medicine, East China Normal University, Shanghai 200241, P.R. China*

*^b^ Faculty of Chemistry and Food Chemistry & Center for Advancing Electronics Dresden, Technische Universität Dresden, Dresden 01062, Germany*

*^c^ Shanghai Key Laboratory of Hydrogen Science & Center of Hydrogen Science, Shanghai Jiao Tong University, Shanghai, 200240, P.R. China*

*^d^ School of Mechanical Engineering, Shanghai Jiao Tong University, Shanghai 200240, P.R. China*

*^e^ Joint Key Laboratory of the Ministry of Education, Institute of Applied Physics and Materials Engineering, University of Macau, Avenida da Universidade, Taipa, Macau SAR 519000, P. R. China*

*^f^* *Siyuan Laboratory, Guangdong Provincial Engineering Technology Research Center of Vacuum Coating Technologies and New Energy Materials, Department of Physics, Jinan University, Guangzhou 510632, P.R. China*

**Corresponding authors*

*E-mail: lkpan@phy.ecnu.edu.cn (Likun Pan); lijinliang@email.jnu.edu.cn (Jinliang Li);* *hao.xu3@tu-dresden.de (Hao Xu)*

**Supplementary Notes**

***Material***

Super P carbon black, hydrophobic polytetrafluoroethylene (PTFE), Ag/AgCl electrode, Pt electrode, Zn foil (0.1 mm, ≥99.9% or 0.01 mm, ≥99.9%), Cu foil (0.02 mm, ≥99.9%), and Ti foil (0.02 mm, ≥99.9%) were all acquired from Shenzhen Kejing Star Technology. By employing an abrasive paper to remove the passivation layer, the Zn foil was produced. Perfluoro-3,6-dioxa-4-methyloct-7-enesulphonyl fluoride (PSVE), ethanol (≥75%), V_2_O_5_ (≥99.0%), ZnSO_4_·7H_2_O (≥99.0%), and NaOH (≥99.5%) were acquired from Sigma-Aldrich Chemical Co. All of the other reagents were analytical grade and didn't require any additional purification. Deionized (DI) water was prepared form the lab.

***Preparation of the electrolyte***

Firstly, 5.75g of ZnSO_4_·7H_2_O were dissolved in the 10 ml of DI, with stirring for 3 hours at a speed of 500 rpm, until the solution is completely clear, which is noted as the 2M ZnSO_4_ (ZS). Secondly, to study the appropriate proportion of the ethanol (EA) and DI, 5.75 g of ZnSO_4_·7H_2_O were dissolved in 10 ml mixed solution of EA and DI and the ratio of EA in the DI solution is 20 vol. %. The 5.75g of ZnSO_4_·7H_2_O were dissolved in the 10 ml mixed solution, which is noted as ZS/EA. Thirdly, the PSVE was solvated in the EA solution as the volume fraction of 0.5, 1, 2 vol. % and then mixed with DI solution under the ratio of 20 vol. % (according to the second step). Then the 5.75g of ZnSO_4_·7H_2_O were dissolved in the 10 ml mixed solution with the additive of PSVE for 0.04, 0.08 and 0.16 mM, which is noted as ZS/PSVE. If there is no special indication, ZS/PSVE generally refers to 0.08 mM PSVE in the manuscript.

***Preparation of*** ***Na_2_V_6_O_16_ (NVO) cathode***

The synthesis process is corroding to the reported study.^1^ In a typical synthesis, 4 mmol of V_2_O_5_ and 4 mmol of NaOH were dissolved in the DI. The volume of the solution was set to 85 mL, and magnetically stirred at room temperature for 1h. The solution was then transferred into an autoclave and heated at 180 °C for 24 h. The sample, Na_2_V_6_O_16_ (NVO) nanowire, was collected by centrifugation, washed with water and alcohol, and then dried at 80 °C overnight.

***Material Characterizations***

Field emission scanning electron microscopy (FESEM, Hitachi S-4800) and energy dispersive X-ray spectroscopy (EDS) mapping were used to analyze the sample morphologies. Laser scanning microscopy (KEYENCE VK-X150) was used to assess the Zn anode's surface roughness and three-dimensional pictures following cycles in various electrolytes. Wavenumbers 4000-400 cm^-1^ were acquired using Fourier transform infrared spectroscopy (FTIR, Thermo Scientific, Nicolet iS50). A Bruker 500 MHz AVANCE III NMR spectrometer was used to gather the nuclear magnetic resonance (NMR) spectra. A 532 nm laser was used to gather Raman spectra of several electrolytes from Horiba HR Evolution. The materials' crystal structure was examined by X-ray diffraction (XRD, Panalytical PRO PW3040/60) at a scanning rate of 2° min-1 using Cu Kα radiation (λ = 1.5406 Å). An X-ray photoelectron spectrometer (XPS, Thermo Escalab 250 Xi) fitted with an Al-Kα X-ray source (1486.6 eV) was used to perform the X-ray photoelectron spectroscopy of the Zn anodes following cycles in various electrolytes.

***Electrochemical measurements***

The coin-type cells (CR2032) were assembled in an open-air environment. First, a 7:2:1 mass ratio was used to combine the resulting NVO sample with PVDF and super P carbon black. The aforementioned mixes were then combined with N-methylpyrrolidone (NMP, 99.9%, Aladdin) to create a slurry, which was evenly applied to the copper foil surface and vacuum-dried for 12 hours at 90 °C. In this investigation, the average mass loading of NVO was around 1-2 mg cm^-2^ for traditional cathodes and 3-5 mg cm^-2^ for low N/P ratio cathodes. Commercial glass fibers (Whatman, GF/C) were posted as the separators, and polished zinc foils were created as the anodes.

Using coin cells of the 2032 type, Zn//Cu, Zn//Zn, and NVO//Zn complete batteries were electrochemically characterized. At -150 mV, the Zn plating/stripping performance of symmetric Zn//Zn cells was assessed using galvanostatic discharge/charge experiments. With varying PSVE contents in the NaSO_4_ electrolytes, the hydrogen evolution reaction potential was measured using the linear sweep voltammetry (LSV) method at a scan rate of 1 mV s^-1^. Ag/AgCl, platinum plate, and zinc foil were used as the reference electrode, counter electrode, and working electrode, respectively. Tafel plots were measured by scanning between -2 and 0 V at 1 mV s^-1^ in the three-electrode system. Zn foil served as the reference electrode and counter electrode in the fabrication of half-cells, whereas Cu foil served as the working electrode. During the charging process, the half-cell's cutoff voltage was set at 0.5 V (vs. Zn/Zn^2+^).

The following equations were used to calculate the transfer number of Zn^2+^, which was determined using Nyquist plots and chronoamperograms (CAs) at a voltage of 5 mV:^2^

τ_zn2+_= 𝐼𝑠(Δ𝑉 - 𝐼_0_𝑅_0_)/ 𝐼_0_ (Δ𝑉 - 𝐼𝑠𝑅𝑠) (1)

where R_0_ and R_s_ represent the resistance prior to and following the CAs test, and I_0_ and I_s_ represent the initial and steady state current. The applied voltage polarization (5 mV) is represented by the Δ𝑉.

The calculation of differential capacitance was followed the Equation:^3^

C=(2π*fZ*_im_)^-1^  (2)

where 𝑓 (Hz) is the impedance test frequency, 1000 Hz was chosen for the experiment, and C (μF cm^-2^) is the electric double layer's differential capacitance. The impedance's imaginary part is represented as Z_im_ (ohm).

EIS experiments using a frequency range of 0.1 Hz to 100,000 Hz were used to determine the ionic conductivities of the electrolytes using stainless steels (SS)//electrolyte//SS using the following equation:^4^

σ=L/(R×A) (3)

where the separator thickness, SS electrode contact area, and bulk resistance (R_s_) are represented by L, A, and R, respectively, based on the EIS plots.

The electric double-layer capacitance was determined using the formula:

C=*i*_c_/*v* (4)

*i*_c_=(i_0_v^+^-i_0_v^-^)/2 (5)

where C is the capacitance, *i*c is the double layer current, and *v* is the scan rate, the slope of the *ic* against *v* graphs is used to calculate capacitance (C). At 0 V, it is the half of the current difference between the forward and negative scans.

Operando X-ray diffraction experiments were performed at beamline P02.1 at the PETRA III synchrotron (DESY, Hamburg, Germany), using a monochromatic X-ray beam with a wavelength of 0.207351 Å. The experiments were carried out using coin cells with Kapton windows. The electrochemical measurement was conducted with a multichannel potentiostat VMP3 (Biologic). Each diffraction pattern was recorded every 10 min, while the cells were charged and discharged at 0.5 A g^-1^. The electrolyte used was 2 M ZS and ZS/PSVE. The NVO and the zinc foil were used as the cathode and the anode.

***Density-Functional theory (DFT)***

For calculations of Zn(002) surface, a 4×4×2 supercell was constructed, respectively. Then the surface was separated by a 15 Å vacuum layer along c axis. The plane wave cutoff energy was set to 550 eV and 1 × 1 × 1 Monkhorst-Pack k-point mesh was set for geometry optimization, and 3 × 3 ×1 for calculation of electronic structure and charge density difference. The convergence criterion is to confirm the total energy converged to 2 meV per atom. For van der Waals correction, the DFT-D3 methodwith Becke-Jonson damping was applied within VASP software.

All quantum calculations, including geometry optimizations, frequency analyses, and molecular electrostatic potential (ESP) mapping, were performed using density functional theory (DFT) with the B3LYP functional and 6-311++G(d,p) basis set, as implemented in Gaussian 16.^5^ The empirical dispersion correction (Grimme's D3 with Becke–Johnson damping) was included by using the keyword empiricaldispersion=gd3bj. Tight SCF convergence (scf=tight) and full population analysis (pop=full) were applied. Frontier molecular orbitals, including HOMO and LUMO, were visualized using the Multiwfn 3.8 program.^6^

***Comsol Simulation***

Utilize Comsol Multphysics to conduct finite element simulation. The Zn negative electrode is shown in the geometric model as the bottom protrusion. The lower border of the geometric model has a grounding boundary condition (E = 0 V), whereas the upper boundary has a constant potential condition (E = 1 V). Simulating the current distribution on the negative electrode surface using the MUMPS steady-state solver. Utilizing the COMSOL Multiphysics software's secondary current distribution and diluted matter transport module to carry out finite element simulation. Electrochemical processes and the ion flux at the electrolyte contact are related, accounting to Faraday's law. The Butler Volmer equation is utilized to calculate the surface reaction kinetics of the lower boundary, while the Zn^2+^ concentration of the upper boundary electrolyte is set at 2 M. The exchange current density is 10 mA cm^-2^. Using the Nernst Planck equation, Zn^2+^ migration induced by an electric field can be explained.


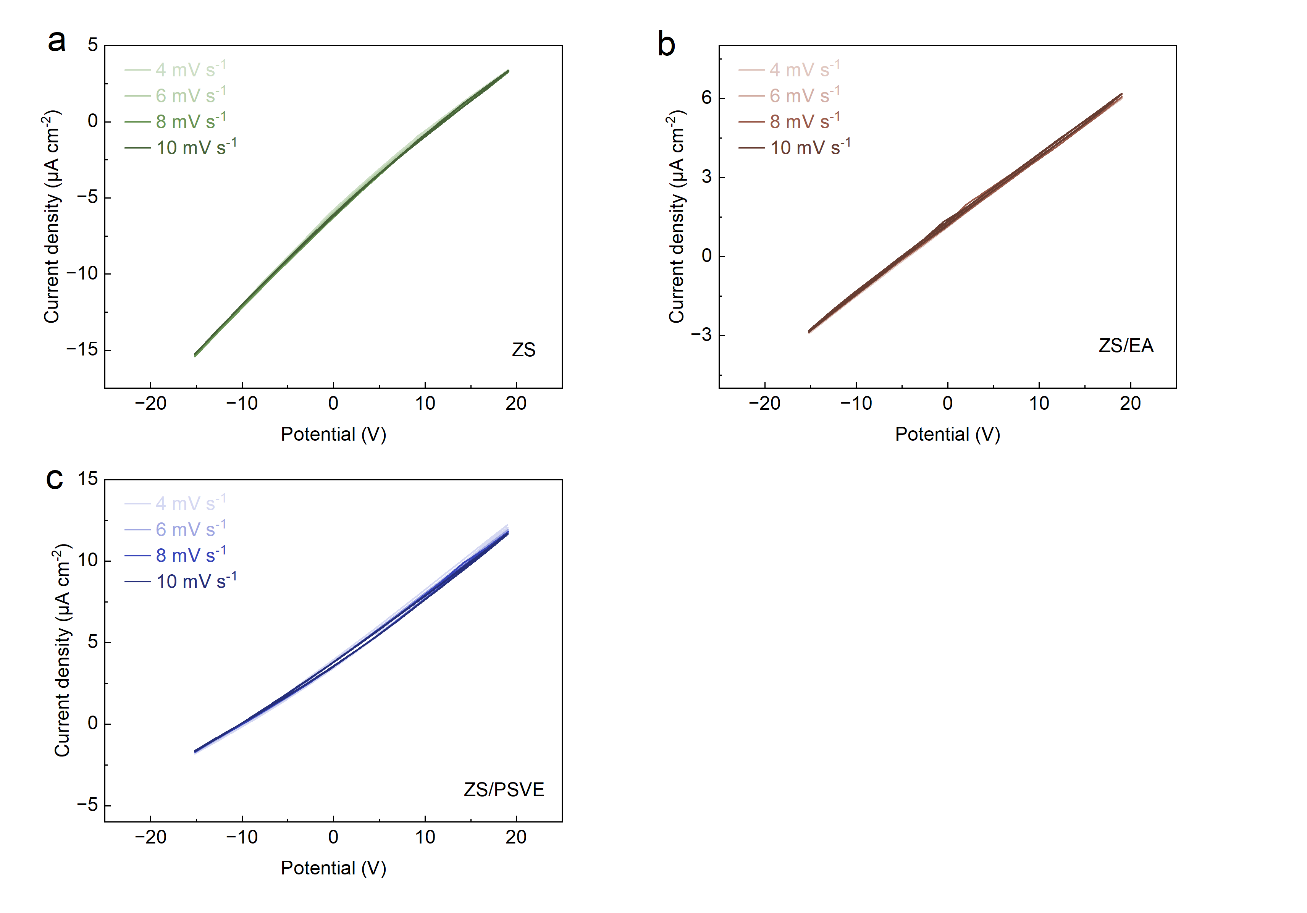


**Figure S1.** The CV curves of the symmetric batteries fabricated by different electrolytes of a) the ZS, b) the EA, and c) the ZS/PSVE.


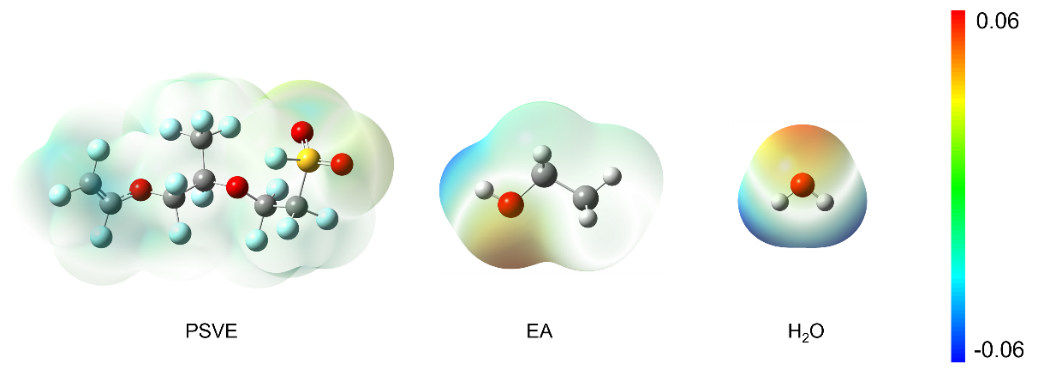


**Figure S2.** Electrostatic potential (ESP) mapping of the PSVE, EA, and H_2_O.


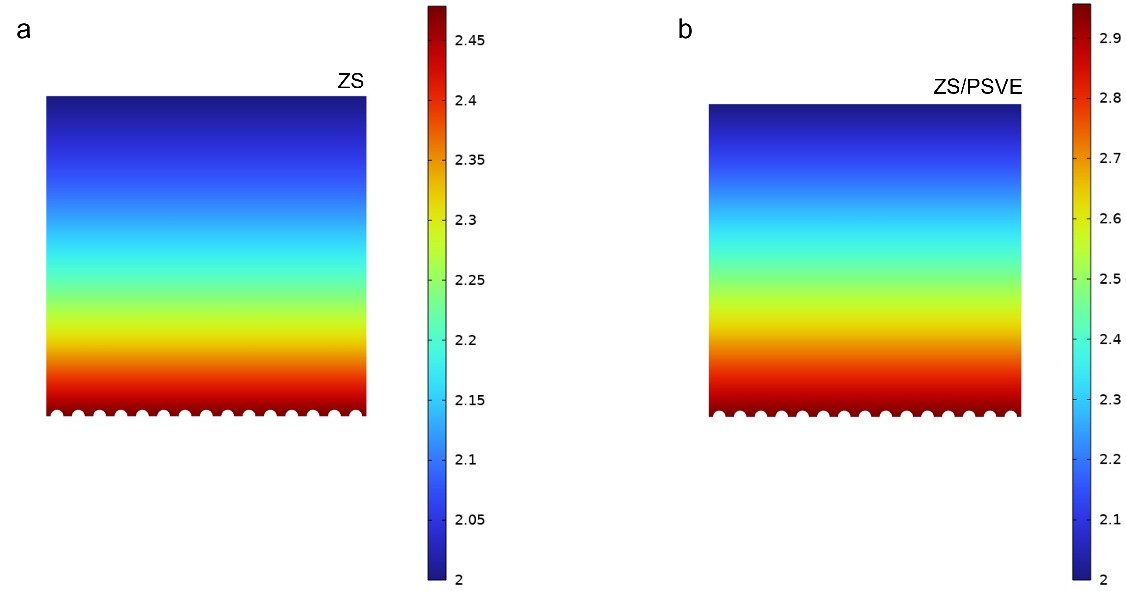


**Figure S3.** Simulated Zn^2+^ concentration distributions on the Zn anode in different electrolytes of a) the ZS and b) the ZS/PSVE electrolyte.


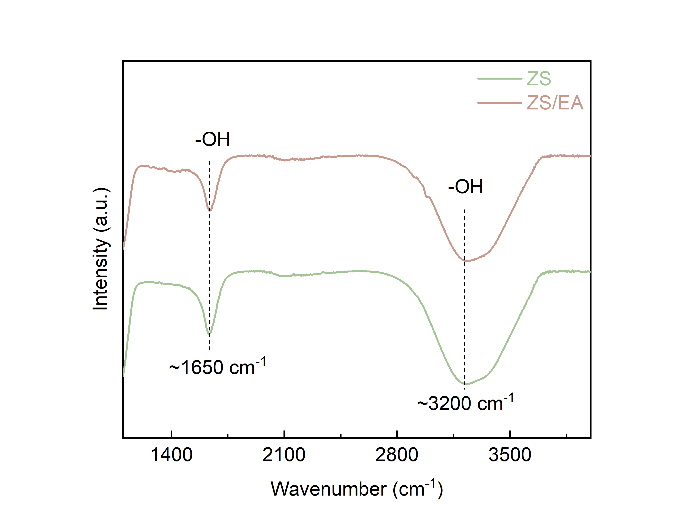


**Figure S4.** The FTIR spectra of the ZS and ZS/EA electrolytes.


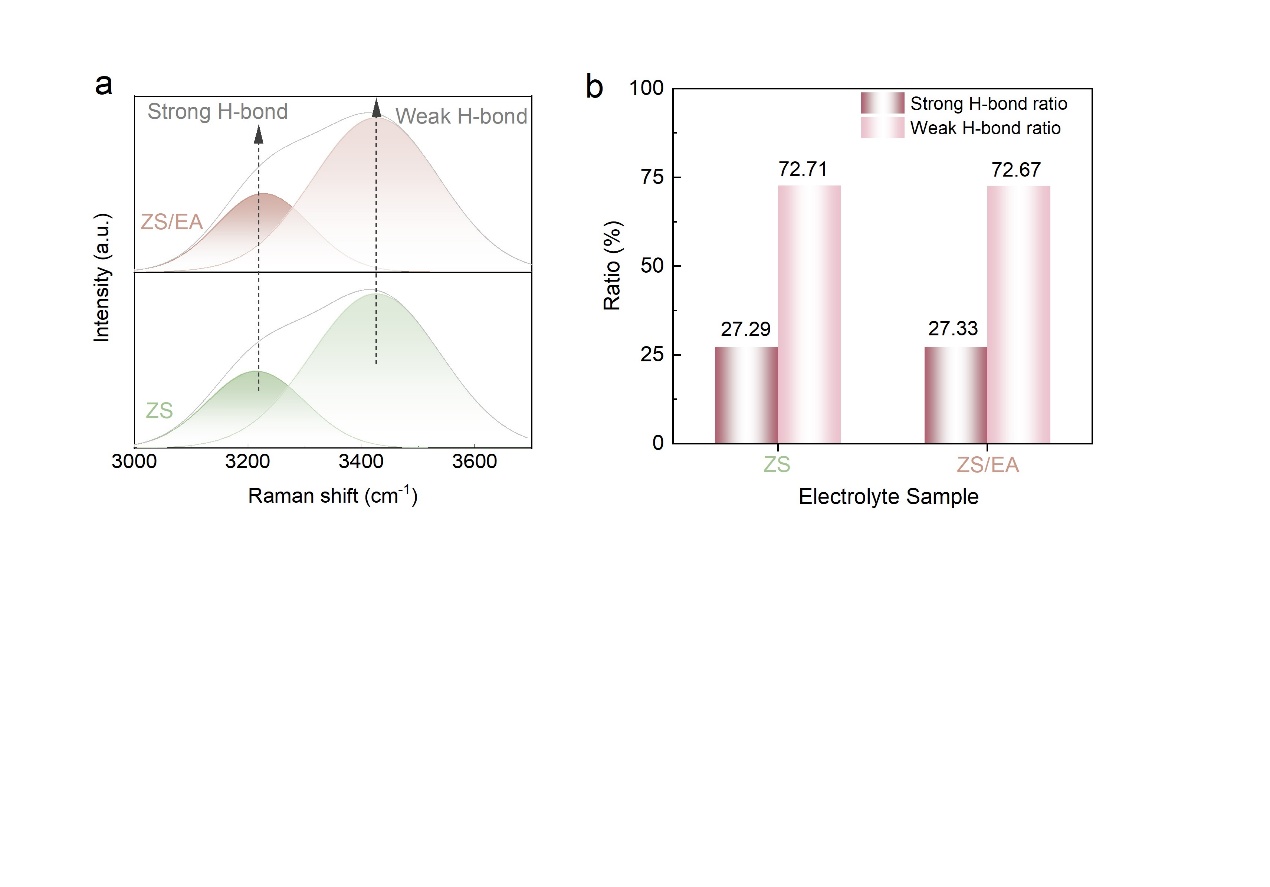


**Figure S5.** a) Raman spectra and b) the corresponding percentages of the strong H-bond and weak H-bond of ZS and ZS/EA electrolytes.


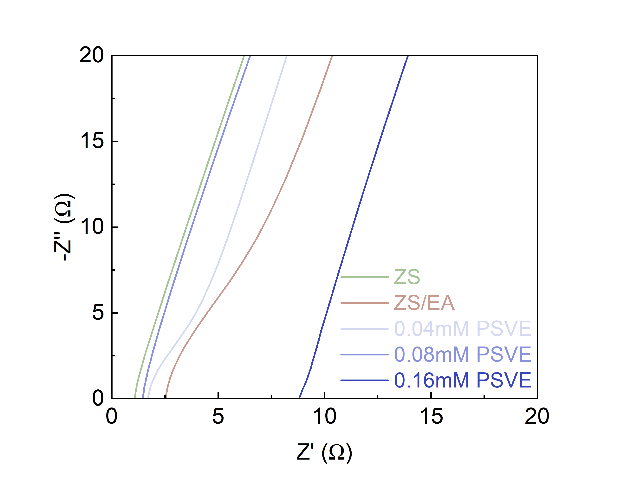


**Figure S6.** The EIS plot of the SS//SS symmetric batteries fabricated by different electrolytes.


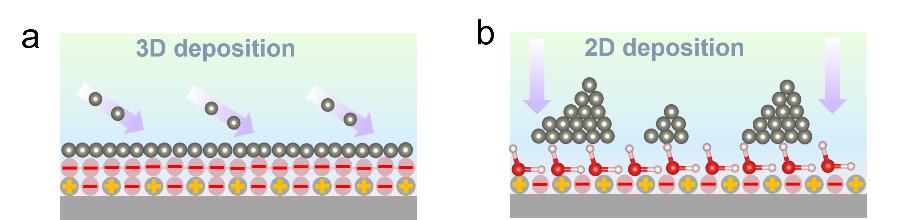


**Figure S7.** The diagram of the different Zinc deposition behavior on the anode at a) 3D deposition and b) 2D deposition.


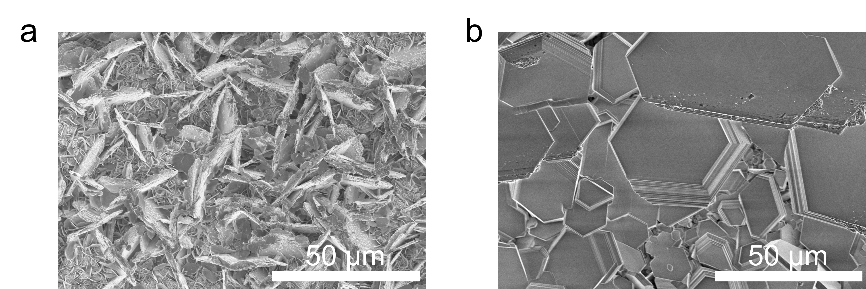


**Figure S8.** The SEM images of the Zn anode surface in a) the ZS electrolyte and b) the PSVE/ZS electrolyte after the CA test.


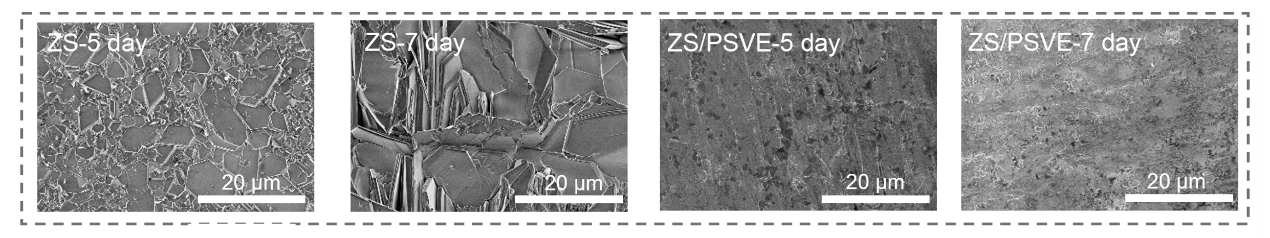


**Figure S9.** The SEM images of Zn foils soaked in the ZS and ZS/PSVE electrolytes after 5 and 7 days.


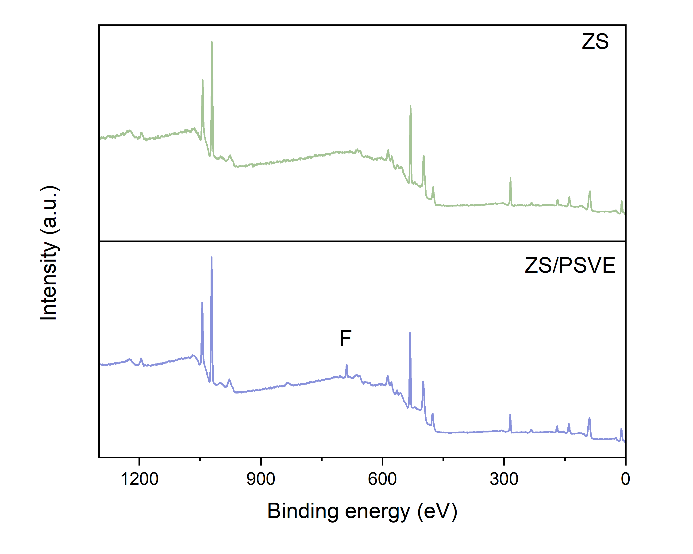


**Figure S10.** The Zn 2p XPS spectra of the Zn anodes in the ZS (up) and ZS/PSVE (down) electrolytes after cycling for 20 cycles.


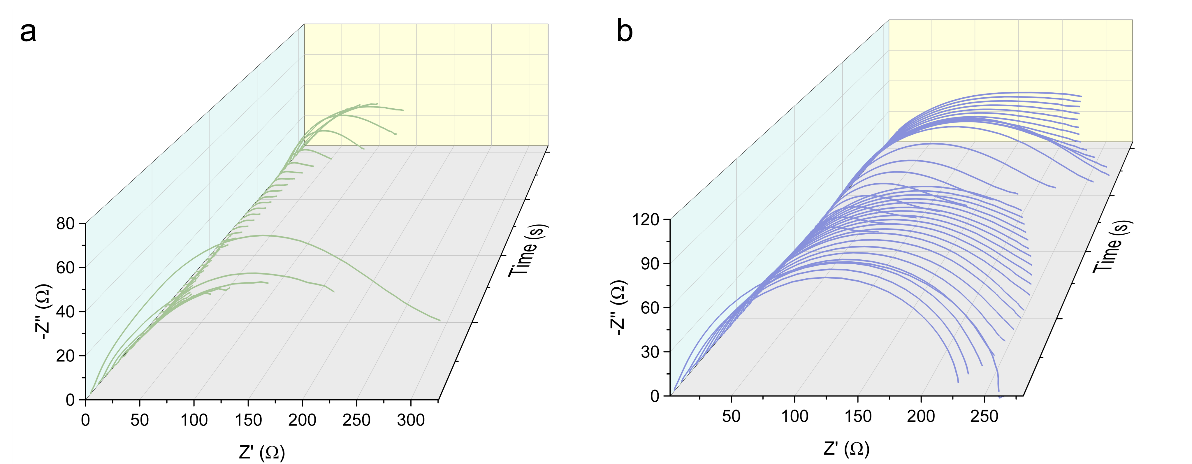


**Figure S11.** The in-situ EIS plot of symmetric Zn//Zn batteries test in the first cycle of charging and discharging with different electrolytes of a) the ZS electrolyte and b) the ZS/PSVE electrolyte.


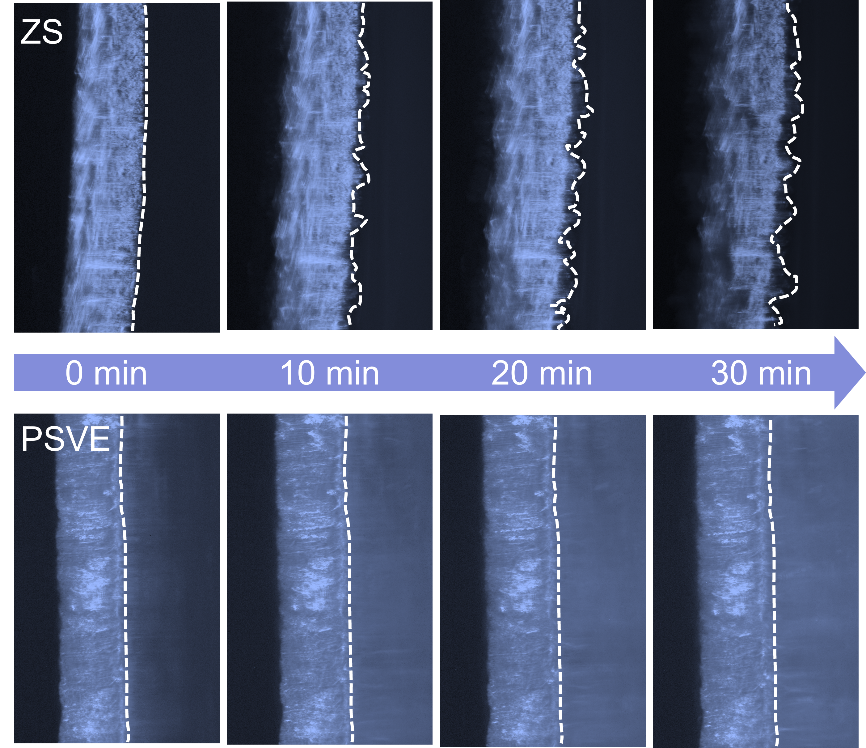


**Figure S12.** The in-situ optical microscopy images of Zn deposition in the ZS electrolyte and the ZS/PSVE electrolyte.


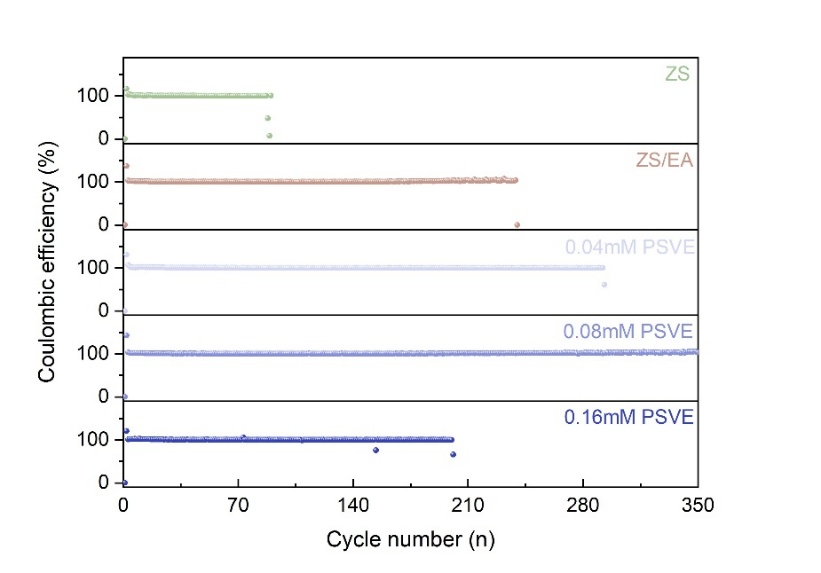


**Figure S13.** The Coulombic efficiency (CE) of Cu//Zn batteries in the different electrolytes with gradient PSVE concentration


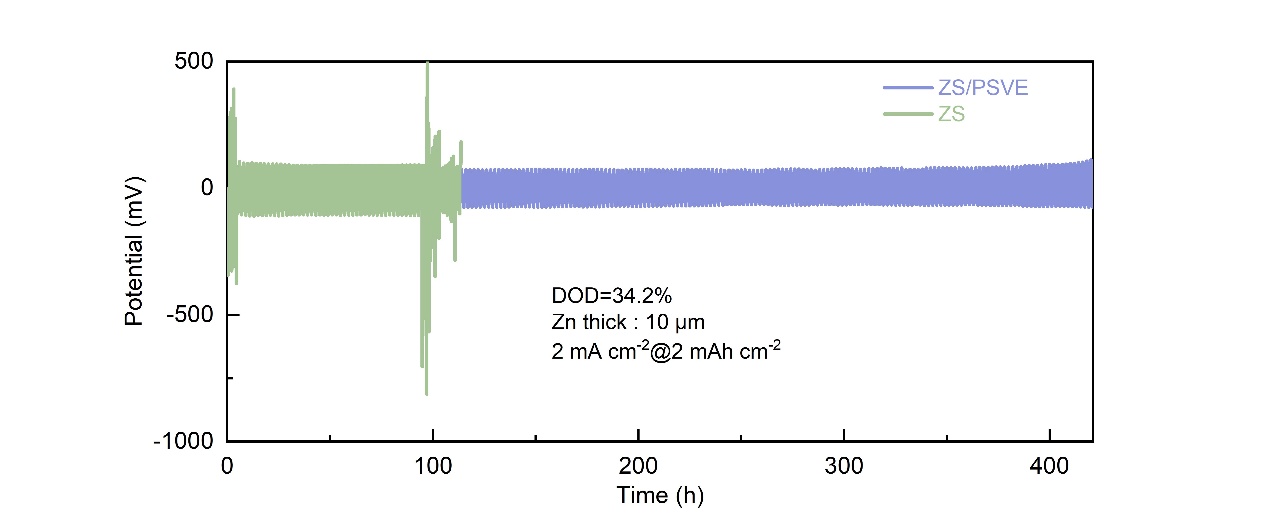


**Figure S14.** The cycling performance of the Zn//Zn batteries assembled with different electrolytes at the DOD of 34.2 %.


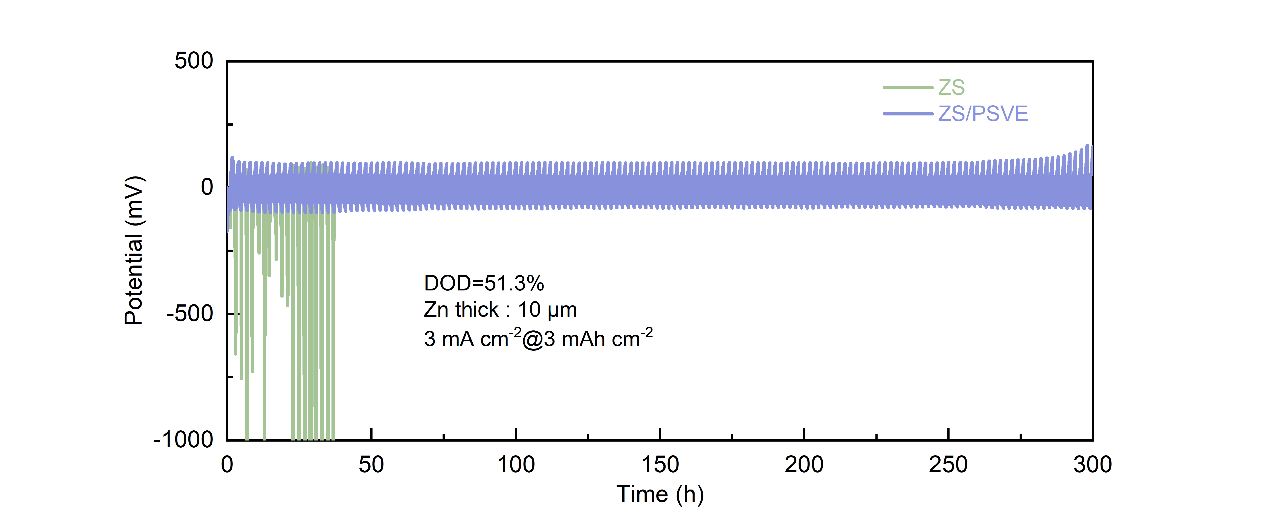


**Figure S15.** The cycling performance of the Zn//Zn batteries assembled with different electrolytes at the DOD of 51.3 %.


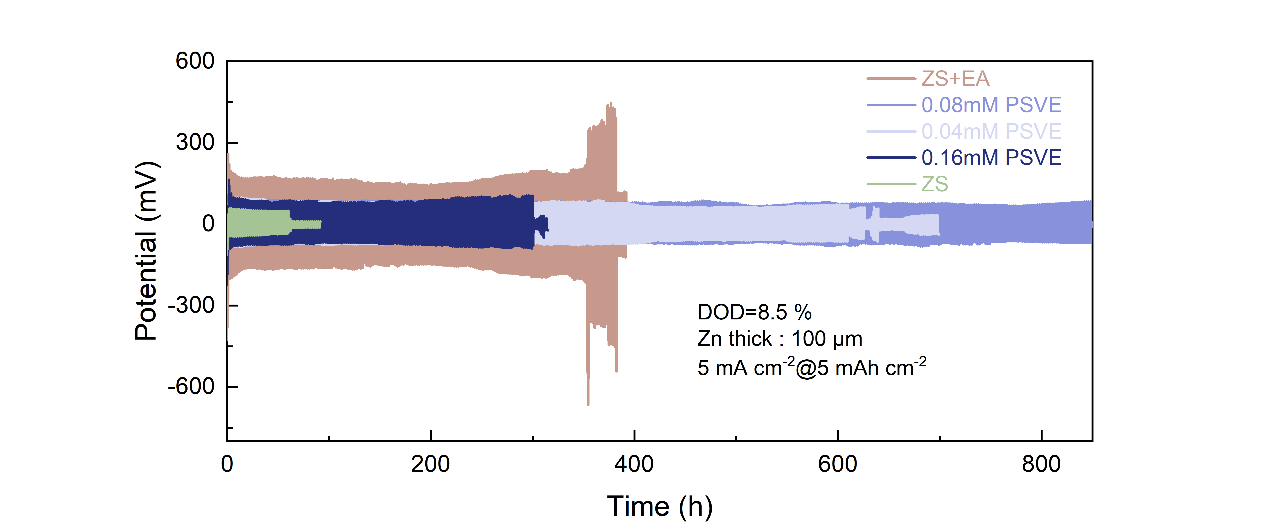


**Figure S16.** The cycling performance of the Zn//Zn batteries assembled with different concentrations of the PSVE additive in the traditional ZS electrolyte at the DOD of 8.5 %.


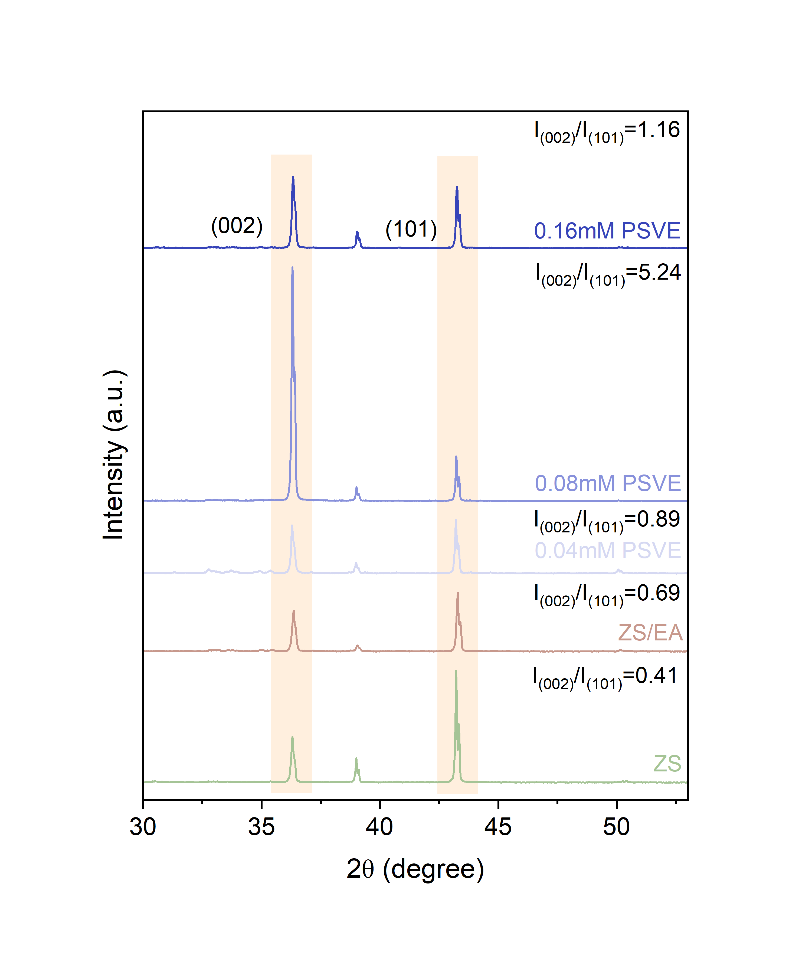


**Figure S17.** The corresponding XRD patterns of the Zn anode after the striping/plating within containing different concentrations of the PSVE electrolytes.


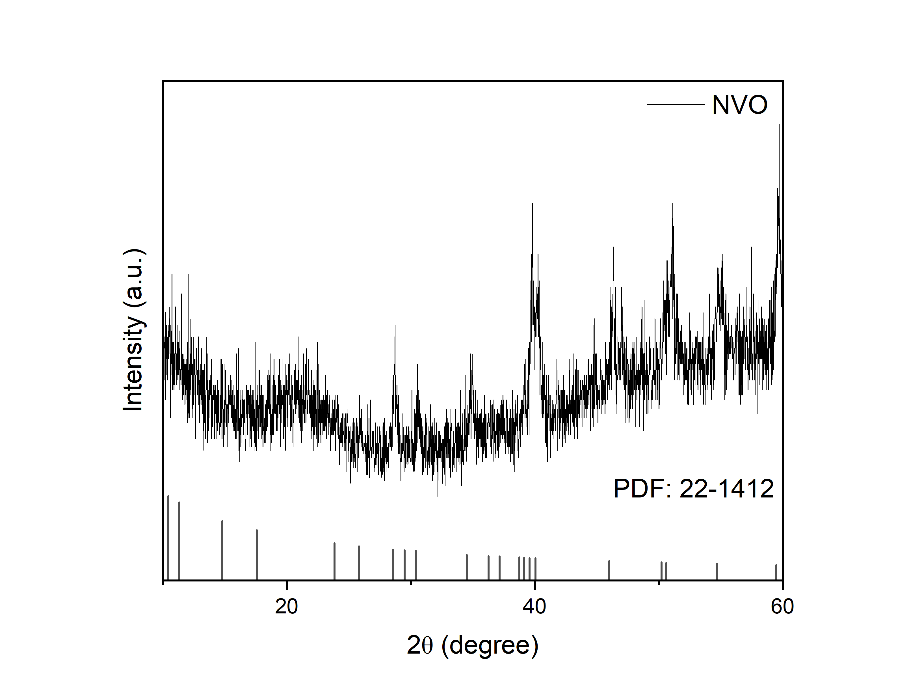


**Figure S18.** The XRD patten of the NVO cathode.


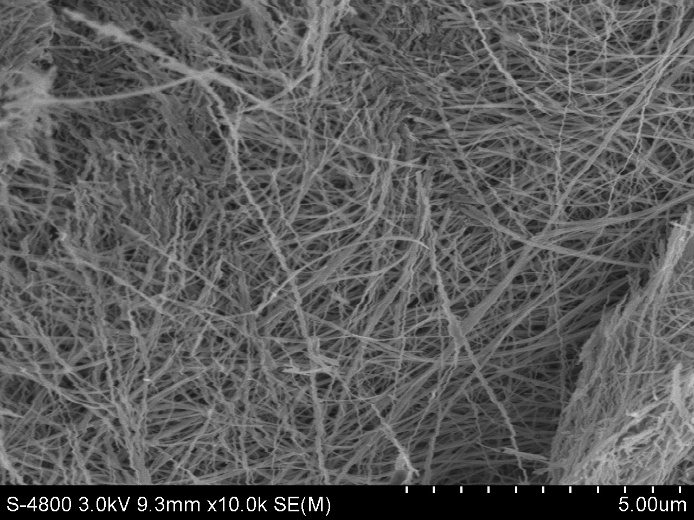


**Figure S19.** The SEM morphologies of the NVO nanowires.


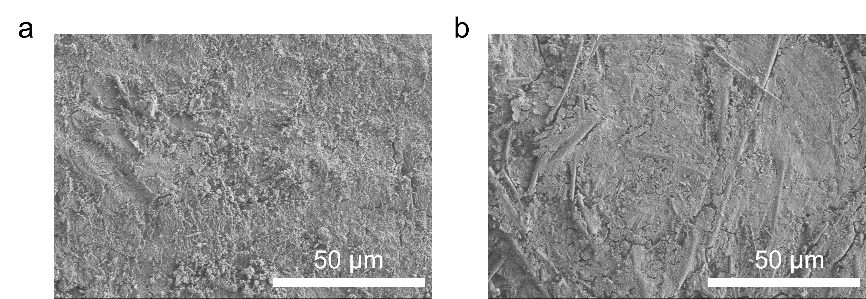


**Figure S20.** The SEM images of the NVO cathode a) original state before 1000 cycled, b) after 1000 cycle in the PSVE-containing electrolyte.


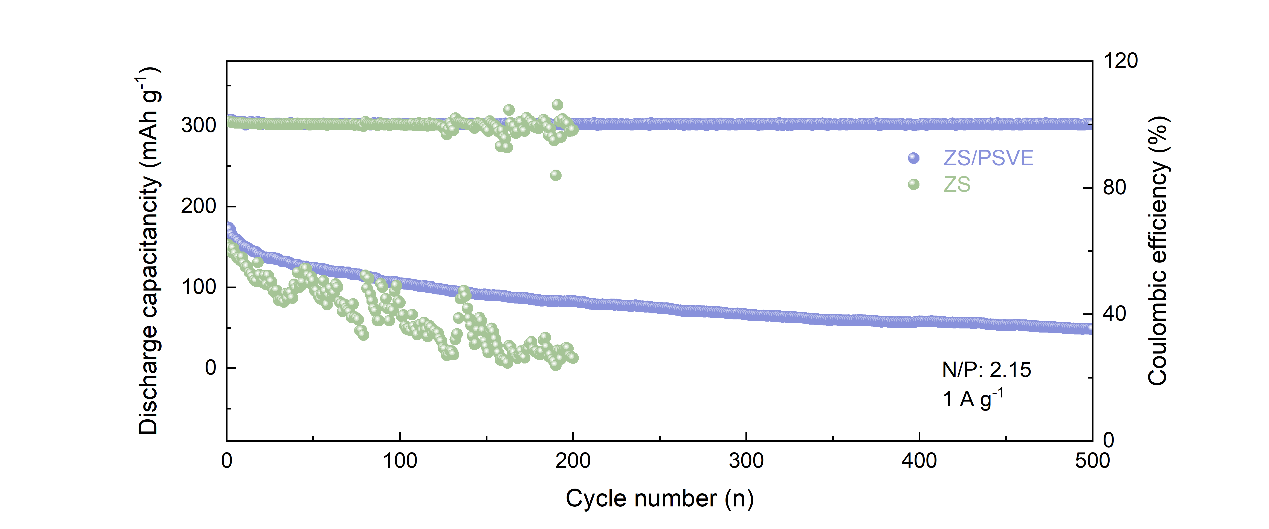


**Figure S21.** The cycle performance of the NVO//Zn full batteries with the ZS and ZS/PSVE electrolytes.

**Table S1.** Comparison of cumulative capacity in this work with other previous reports.

| Electrolyte | Working condition (mA cm^-2^ @mAh cm^-2^) | Lifespan (h) | DOD (%) | Ref. |
| --- | --- | --- | --- | --- |
| 2 M ZnSO_4_+0.05 M KPF_6_ | 3@3  4.7@4.7 | 250  120 | 50  80 | ^7^ |
| 2 M Zn(OTF)2+25 Mm Zn(H_2_PO_4_)_2_ | 1@1  5@1  5@5 | 1200  220  65 | 2.8  2.8  14.2 | ^8^ |
| 1 M ZnSO_4_+1 wt% polyamino acid | 5@3 | 300 | 5.1 | ^9^ |
| 1 M ZnSO_4_+0.5 M acesulfame potassium | 40@20 | 220 | 67.6 | ^10^ |
| 2 M ZnSO_4_+0.1 M  D-Arabinose | 1@40 | 250 | 76.6 | ^11^ |
| 2 M ZnSO_4_+5 %  trifluoroacetamide | 40@5  6@3 | 250  76 | 51.2  51.2 | ^12^ |
| 1 M ZnSO_4_+0.05 M sodium glycerophosphate (SG) | 1@1 | 1000 | 1.7 | ^13^ |
| 2 M ZnSO_4_+0.5 g L^-1^  saccharin | 10@10 | 550 | 24.4 | ^14^ |
| 2 M ZnSO_4_+300 mM L-glutamine | 5@5  10@10 | 1600  1200 | 8.5  17.4 | ^15^ |
| 2 M ZnSO_4_+1 g L^-1^  Dibenzenesulfonamide | 10@10  32@4.5 | 1100  260 | 21.4  9.6 | ^16^ |
| 2 M ZnSO_4_+ 10 mM acesulfame | 2@1 | 270 | 50 | ^17^ |
| This work | 2@2  3@3  4@4  5@5 | 420  300  260  210 | 34.2  51.3  68.4  85.5 |  |

**Reference**

1. P. Hu, T. Zhu, X. Wang, X. Wei, M. Yan, J. Li, W. Luo, W. Yang, W. Zhang, L. Zhou, Z. Zhou and L. Mai, *Nano Lett*. **2018**, *18*, 1758-1763.

2. P. Ruan, S. Liang, B. Lu, H. J. Fan and J. Zhou, *Angew. Chem., Int. Ed.* **2022**, *61*, e202200598.

3. Q. Wang, F. Zhang, Y. Shu, H. Xiao, X. Zhang, X. Ma, J. Liu, Y. Wang, J. Huang and Y. Xia, *Angew. Chem., Int. Ed.* **2025**, *64*, e202418928.

4. H. Ren, S. Li, B. Wang, Y. Zhang, T. Wang, Q. Lv, X. Zhang, L. Wang, X. Han, F. Jin, C. Bao, P. Yan, N. Zhang, D. Wang, T. Cheng, H. Liu and S. Dou, *Adv. Mater.* **2023**, *35*, e2208237.

5. T. Lu, F. Chen, *J. Comput. Chem.* **2012**, 33, 580-592.

6. T. Lu, *J. Chem. Phys.* **2024,** 161, 082503.

7. Y. Chu, S. Zhang, S. Wu, Z. Hu, G. Cui and J. Luo, *Energy Environ. Sci.* **2021**, *14*, 3609-3620.

8. X. Zeng, J. Mao, J. Hao, J. Liu, S. Liu, Z. Wang, Y. Wang, S. Zhang, T. Zheng, J. Liu, P. Rao and Z. Guo, *Adv. Mater.* **2021**, *33*, e2007416.

9. J. Liu, W. Song, Y. Wang, S. Wang, T. Zhang, Y. Cao, S. Zhang, C. Xu, Y. Shi, J. Niu and F. Wang, *J. Mater. Chem. A* **2022**, *10*, 20779-20786.

10. G. Qu, Y. Zhao, C. Li, Y. Zhai, Y. Kong, X. He, L. Kong, C. Wang, M. Chen, K. Song, Z. Liu and L. Xu, *Angew. Chem., Int. Ed.,* **2025,** *64*, 202422036.

11. Y. Yang, Y. Li, Q. Zhu and B. Xu, *Adv. Funct. Mater.* **2024**, *34*, 2316371.

12. M. Wu, X. Wang, F. Zhang, Q. Xiang, Y. Li and J. Guo, *Energy Environ. Sci.* **2024**, *17*, 619-629.

13. J. Hao, L. Yuan, Y. Zhu, M. Jaroniec and S. Z. Qiao, *Adv. Mater.* **2022**, *34*, e2206963.

14. C. Huang, X. Zhao, S. Liu, Y. Hao, Q. Tang, A. Hu, Z. Liu and X. Chen, *Adv. Mater.* **2021**, *33*, e2100445.

15. T. Yan, S. Liu, J. Li, M. Tao, J. Liang, L. Du, Z. Cui and H. Song, *ACS Nano*, **2024**, *18*, 3752-3762.

16. Y.-X. Song, J. Wang, X.-B. Zhong, K. Wang, Y.-H. Zhang, H.-T. Liu, L.-X. Zhang, J.-F. Liang and R. Wen, *Energy Stor. Mater.* **2023**, *58*, 85-93.

17. Q. Guan, J. Li, L. Li, P. Chai, Y. Li, S. Zhang, X. Yu, L. Bao, J. Peng and X. Li, *Chem. Eng. J.* **2023**, *476*, 146534.
